# Metalloenzyme signatures in authigenic carbonates from the Chukchi Borderlands in the western Arctic Ocean

**DOI:** 10.1038/s41598-022-21184-6

**Published:** 2022-10-05

**Authors:** Dong-Hun Lee, Jung-Hyun Kim, Yung Mi Lee, Germain Bayon, Dahae Kim, Young Jin Joe, Xudong Wang, Kyung-Hoon Shin, Young Keun Jin

**Affiliations:** 1 Department of Marine Sciences and Convergent Technology, Hanyang University ERICA Campus, Ansan, 15588 Republic of Korea; 2 Korea Polar Research Institute, Incheon, 21990 Republic of Korea; 3 CNRS, Ifremer, Brest University, Geo-Ocean, 29280 Plouzané, France; 4 Shanghai Engineering Research Center of Hadal Science and Technology, College of Marine Sciences, Shanghai Ocean University, Shanghai, 201306 China; 5 Present Address: Marine Environment Research, Division, National Institute of Fisheries Science, Busan, Republic of Korea

**Keywords:** Biogeochemistry, Environmental sciences

## Abstract

Migration of methane-rich fluids at submarine cold seeps drives intense microbial activity and precipitation of authigenic carbonates. In this study, we analyzed microbially derived authigenic carbonate samples recently recovered from active gas hydrate mounds on the southwestern slope of the Chukchi Borderlands (CB), western Arctic Ocean. Our main aim was to characterize the distribution patterns of trace elements in carbonate-hosted lipid fractions to assess metalloenzyme requirements of microbes involved in anaerobic oxidation of methane (AOM). We measured stable isotopes, trace elements, lipid biomarkers, and genomic DNA, and results indicate the dominance of AOM-related lipid biomarkers in studied carbonate samples, as well as a predominant occurrence of the anaerobic methanotrophic archaea (ANME)-1. We also report evidence for significant preferential enrichments of various trace elements (Li, Ni, Co, Cu, Zn, and Mo) in the total lipid fractions of CB carbonates, relative to elemental compositions determined for corresponding carbonate fractions, which differ from those previously reported for other seep sites. We hypothesize that trace element enrichments in carbonate-hosted lipid fractions could vary depending on the type of AOM microbial assemblage. Additional work is required to further investigate the mechanisms of lipid-bound trace elements in cold seep carbonates as potential metalloenzymes in AOM.

## Introduction

Cold seeps occur at ocean margins worldwide^1,2^, corresponding to methane-rich fluid emanations from the seabed into the water column^3,4^. The seepage rates of reduced fluids can vary substantially at the seafloor^5^, leading to a large spectrum of redox conditions in subsurface sediments at cold seeps^6^. A key biogeochemical process in submarine methane seeps is the anaerobic oxidation of methane (AOM), which efficiently consumes a substantial fraction of methane released into the overlying water column^7,8^. During AOM, alkalinity levels strongly increase in the surrounding pore waters because of the production of biocarbonate (HCO_3_^−^), promoting carbonate supersaturation and precipitation^2,9^. Accordingly, authigenic carbonate precipitation is typically encountered in cold seeps^10,11^, which can serve as an archive for investigating methane release events in both modern and ancient seeps^12,13^.

AOM is typically mediated by a consortium of anaerobic methanotrophic archaea (ANME) and sulfate-reducing bacteria (SRB)^14,15^. However, recent studies have also reported that ANMEs can also use other electron acceptors, such as manganese (Mn)- and iron (Fe)-rich oxyhydroxides, to promote methane oxidation in cold seeps^16^. Notably, other trace metals such as nickel (Ni), cobalt (Co), molybdenum (Mo), tungsten (W), and zinc (Zn) can also be involved in AOM, acting as enzymatic co-factors^17,18^. Most of these findings have been obtained from culture experiments^17,19^. A recent study characterized, for the first time, the trace element geochemistry of the total lipid fractions preserved in authigenic carbonates from various seeps worldwide, such as the Congo, Nile deep-sea, and Niger fans, and the Gulf of Mexico^20^, suggesting that such an approach could be used to identify trace metals essential to microbial activity in cold seeps. A previous study reported marked enrichment of Ni, Co, Mo, and W in carbonate-hosted lipid fractions, which were linked to previously identified enzymatic pathways involved in AOM^20^. Nonetheless, information about how AOM may be influenced by changes in trace metal bioavailability in cold seeps remains scarce.

Recently, gas hydrate mound structures have been discovered in the Chukchi Borderlands (CB) of the western Arctic Ocean^21^. Geochemical information on the origin of the emitted gas and pore fluid properties was obtained within this mound structure during the R/V *ARAON* expedition ARA09C in 2018^22^. However, thus far, biogeochemical signatures preserved in authigenic carbonates have not been investigated in these newly discovered gas hydrate mounds. In this study, we provide a detailed geochemical characterization of a series of authigenic carbonate samples using oxygen and carbon stable isotopes and mineralogy, as well as trace element abundances, lipid biomarkers, and nucleic acids. The main objectives of this study were to i) evaluate key environmental factors controlling carbonate precipitation in the CB mounds, ii) characterize geochemical and microbial community characteristics in the CB mounds, and iii) assess the relation of trace metals in microbial activity associated with AOM by comparing our new data for CB geochemical and microbial signatures with those previously obtained at other seeps worldwide^20^. We aimed to test the hypothesis that specific trace element enrichments in the total lipid fractions extracted from authigenic carbonates could be indicative of preferential trace metal utilization by different ANME communities. Our study sheds new light on the metalloenzyme requirements of microbes involved in AOM.

## Results

### Mineralogy and stable isotopic compositions

**Figure 1:**
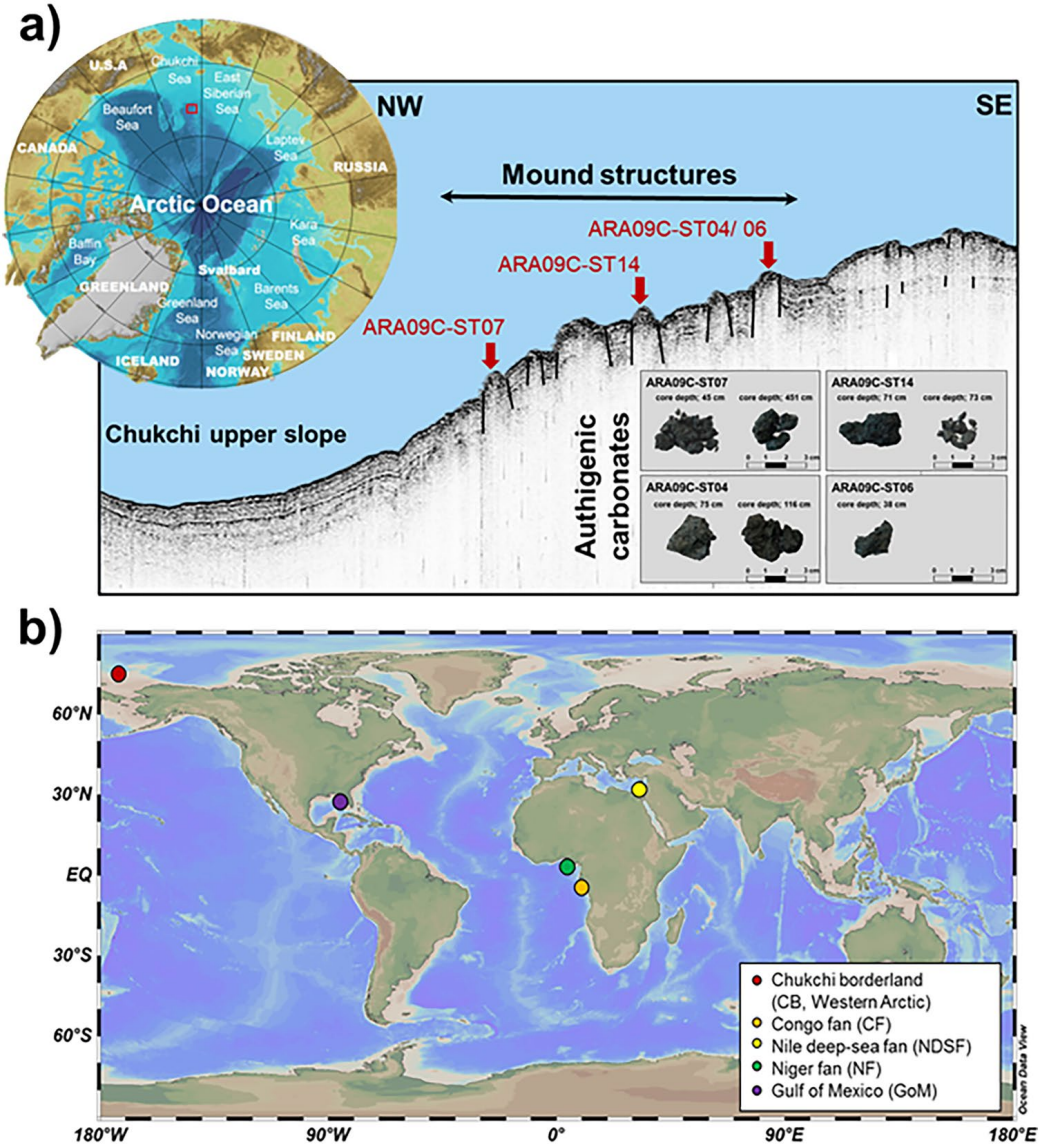
Map showing (**a**) the mound structures discovered in the Chukchi Borderlands (CB) with authigenic carbonates investigated in this study and (**b**) four comparison study sites: Congo fan (CF), Nile deep-sea fan (NDSF), Niger fan (NF), and Gulf of Mexico (GoM). The bathymetric map was generated with the International Bathymetric Chart of the Arctic Ocean Version 3.0 (https://www.ngdc.noaa.gov/mgg/bathymetry/arctic). The sub-bottom profile (SBP) image was acquired using a SBP120 equipment (frequency; 12 kHz, wide-beam angle; 90–120°, Kongsberg). The global ocean map was generated with the Ocean Data View version 5.1.4 (GlobalHR, https://odv.awi.de).

The authigenic carbonate samples collected in this study (Fig. 1, see also Fig. S1 in the Supplementary Information) were dominated by calcite, with a minor contribution from dolomite (Table 1). The δ^13^C values of carbonates (δ^13^C_carbonate_) were depleted, varying between − 37.0 and − 32.8‰. The δ^18^O values of carbonates (δ^18^O_carbonate_) ranged from 3.9 to 5.6‰, with more enriched values occurring in samples from the deeper sediment layers (Table 1). The measured δ^13^C_carbonate_ and δ^18^O_carbonate_ values of bulk CB carbonates partially overlapped with those determined for a series of seep carbonate samples previously investigated^20^ (Table 1 and Fig. S2^10,23–27^).

**Table 1:** Site information and mineral and stable isotopic compositions of authigenic carbonate samples considered in this study.

Sites	Sample names	Sample ID	Location		Water depth (m)	Core depth (cm)	Carbon and oxygen isotopes		Mineral compositions			Reference
			Longitude	Latitude			δ^13^C (VPDB)	δ^18^O (VPDB)	Arag. (wt %)	Cal. (wt %)	Dolo. (wt %)	
Chukchi borderland (Western Arctic)	ARA09C_ST07_GC03 45	CB-1	169° 47.6843' W	75° 42.7173' N	699	45	− 35.7	5.2	n.d	25	n.d	This study
	ARA09C_ST07_GC03 451	CB-2	169° 47.6843' W	75° 42.7173' N		451	− 33.3	3.9	n.d	23	n.d	This study
	ARA09C_ST14_GC03 71–74	CB-3	169° 44.3467′ W	75° 40.3835′ N	646	71	− 37.0	5.2	n.d	24	3	This study
	ARA09C_ST14_GC03 73	CB-4	169° 44.3467′ W	75° 40.3835′ N		73	− 33.3	5.4	n.d	23	3	This study
	ARA09C_ST04_GC04 75–80	CB-5	169° 44.2104' W	75° 40.7934' N	605	75	− 34.8	5.6	n.d	32	6	This study
	ARA09C_ST04_GC03 116–123	CB-6	169° 44.2104' W	75° 40.7934' N	605	116	− 32.8	5.0	n.d	22	6	This study
	ARA09C_ST06_GC01 38	CB-7	169° 44.1945' W	75° 40.8402' N	611	38	− 35.1	5.5	n.d	46	n.d	This study
Congo fan (CF)	ZR2-PL13-P04	CF-1	–	-	2830	0	− 58.5	5.3	n.d	> 50	n.d	Pierre and Fouquet^25^
	ZR2-PL14-P05	CF-2	–	–	3150	0	− 53.6	5.6	n.d	> 50	n.d	Pierre and Fouquet^25^
	BZ1-GBT3-PL7-83	CF-3	–	–	3150	0	− 49.5	2.9	n.d	> 50	n.d	Pierre and Fouquet^25^
Nile deep-sea fan (NDSF)	NL4-CC1	NDSF-1	–	–	3032	0	− 38.9	2.8	72	4	1	Gontharet et al.^24^
	NL7-CC1	NDSF-2	–	–	1691	0	− 41.8	4.2	n.d	51	27	Gontharet et al.^24^
	NL14-CC5	NDSF-3	–	–	2129	0	− 29.2	3.2	70	20	n.d	Gontharet et al.^24^
	NL20-CC1	NDSF-4	–	–	3018	0	− 38.4	3.1	82	5	n.d	Gontharet et al.^24^
Niger fan (NF)	N1-KS-07	NF-1	–	–	1633	0	− 45.9	4.0	87	12	n.d	Rongemaille et al.^27^
	N1-KS-22	NF-2	–	–	1150	300	− 27.8	4.9	90	10	n.d	Rongemaille et al.^27^
	N1-KSF-45	NF-3	–	–	1546	185	− 47.1	6.1	n.d	6	87	Rongemaille et al.^27^
	N1-KI-47	NF-4	–	–	1540	12	− 48.0	5.7	n.d	8	86	Rongemaille et al.^27^
Gulf of Mexico (GoM)	#2 4173-2 (AT340)	GoM-1	–	–	2216	0	− 54.2	4.0	63	n.d	n.d	Feng et al.^10^, Roberts et al.^26^, Feng and Roberts^23^
	#7 4174-2 (GC600)	GoM-2	–	–	1250	0	− 21.4	4.8	n.d	52	n.d	Feng et al.^10^, Roberts et al.^26^, Feng and Roberts^23^
	#65 271-1 (MC462)	GoM-3	–	–	973	0	− 40.5	4.6	80	n.d	n.d	Feng et al.^10^, Roberts et al.^26^, Feng and Roberts^23^
	#67 272-1 (GC415)	GoM-4	–	–	1110	0	− 38.2	3.4	n.d	68	1	Feng et al.^10^, Roberts et al.^26^, Feng and Roberts^23^
	#68 273-1 (GC852)	GoM-5	–	–	1633	0	− 40.2	3.7	n.d	70	1	Feng et al.^10^, Roberts et al.^26^, Feng and Roberts^23^
	#69 273-2 (GC852)	GoM-6	–	–	1633	0	− 40.1	3.7	n.d	62	2	Feng et al.^10^, Roberts et al.^26^, Feng and Roberts^23^
	#70 273-3 (GC852)	GoM-7	–	–	1633	0	− 48.1	4.5	n.d	54	2	Feng et al.^10^, Roberts et al.^26^, Feng and Roberts^23^

n.d. denotes ‘not determined’. ‘–’ indicates ‘not available'.

### Trace element compositions

**Figure 2:**
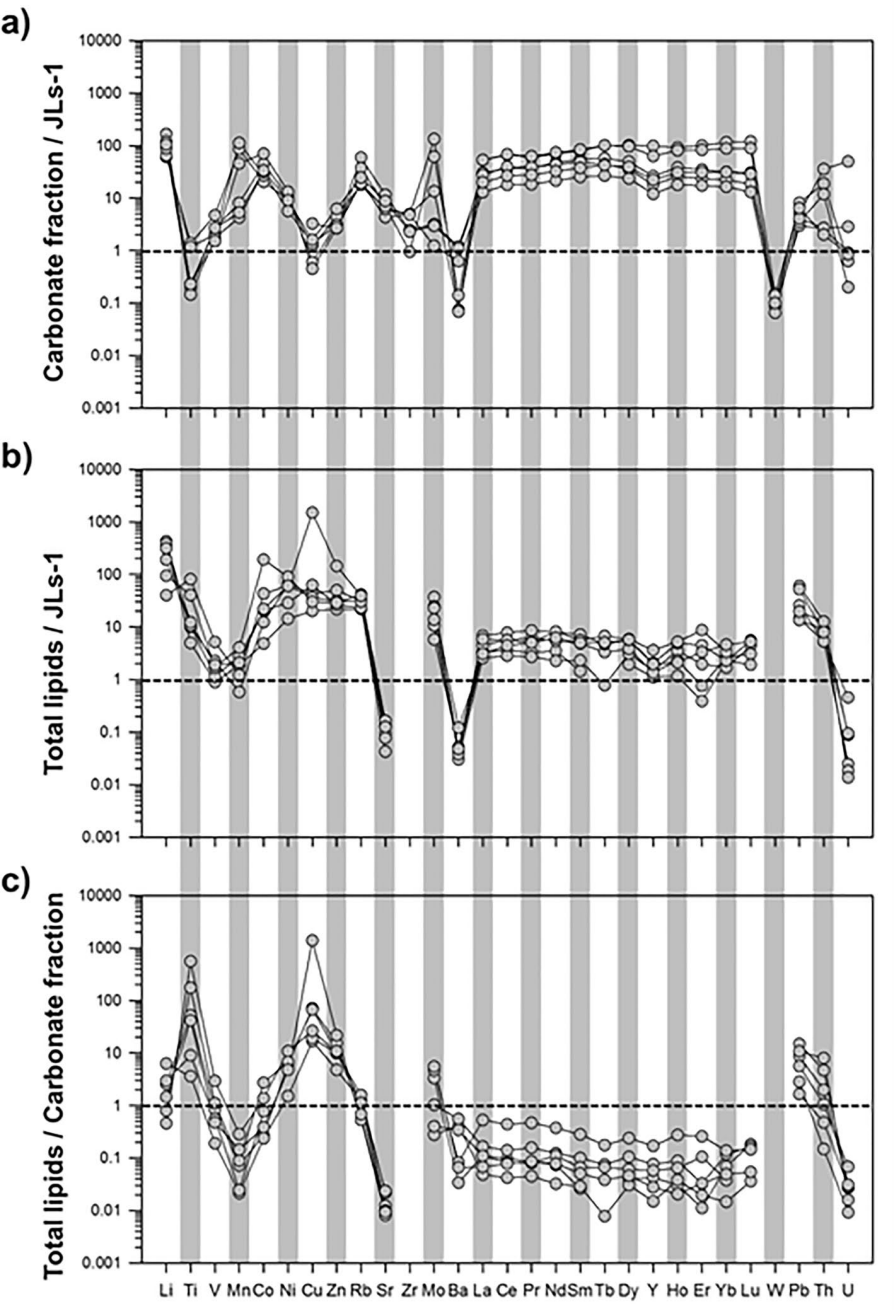
Enrichment factors of (**a**) carbonate fraction (1 M AA leachates) data normalized to values for the JLs-1 carbonate reference material, (**b**) total lipid fraction data normalized to values for the JLs-1 carbonate reference material, and (**c**) total lipid fraction data normalized to values for the corresponding carbonate fractions (1 M AA leachates).

Trace element data for carbonate (1 M AA leachates), sulfide (3 M HNO_3_ leachates), detrital silicate (HF-HCl digestion), and lipid fractions are reported in Tables S1–S4 in the Supplementary Information. Compared to JLs-1 (carbonate reference material; Triassic limestone), the carbonate fractions investigated in this study were generally characterized by much higher trace element abundances (Fig. 2 and Fig. S3), many of which (lithium (Li), transition metals, strontium (Sr), REE, lead (Pb), and thorium (Th)) were enriched up to a few hundred times. In contrast, three elements (titanium (Ti), barium (Ba), and W) showed relatively lower concentrations (up to tenfold depletion compared to JLs-1).

Trace element data for the extracted sulfide-rich solid phases (i.e., 3 M HNO_3_ leachates) were normalized to the data for corresponding carbonate fractions, indicating that some elements (Ti, copper (Cu), zirconium (Zr), and Th) in the carbonate and sulfide fractions were enriched as much as 10 to 100 times (Fig. S3). Trace element abundances in the detrital silicate fractions were also normalized to the Post Archean Australian Shale (PAAS) reference values^28^. Some elements, such as Li, Rb (rubidium), Ba, W, Pb, Th, and U, were relatively enriched in the detrital silicate fractions relative to PAAS (Fig. S3). For the lipid fractions, several trace elements displayed a large range of abundance values (Li: 2.8–29.9 ppm, transition metals: 0.5–300 ppm, Rb: 2.2–4.1 ppm, Sr: 12.0–45.0 ppm, Ba: 13.0–22.0 ppm, Tables S1–S4). In contrast, REEs displayed very low abundances (< 1.34 ppm) in the lipid fractions. Notably, the lipid fraction data normalized to JLs-1 and the carbonate fractions of the CB carbonates clearly showed marked enrichment of Ti, Ni, Cu, Zn, and Pb (as much as 10–1000 times; Fig. 2, see also Fig. S4).

### Lipid biomarker characteristics

**Figure 3:**
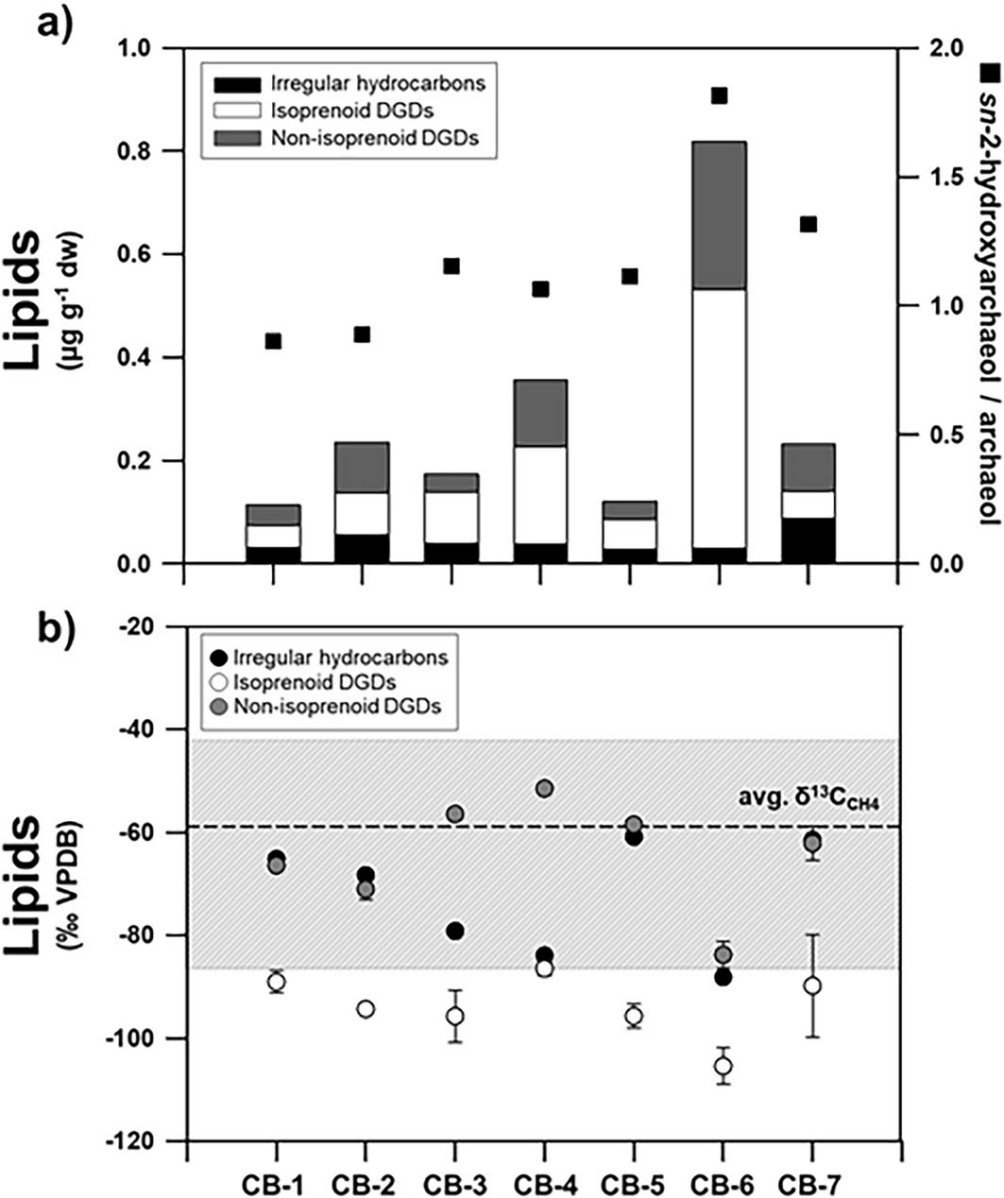
Lipid biomarker signatures of authigenic carbonates from the CB: (**a**) concentrations of major lipid biomarkers and ratios of *sn*-2-hydroxyarchaeol relative to archaeol, and (**b**) carbon isotopic compositions of major lipid biomarkers. Note that the dashed line indicates the average value of δ^13^C of methane (δ^13^C_CH4_) while the shaded area shows the full range of δ^13^C_CH4_ values.

Concentrations of archaeal and bacterial lipids showed large variations in the CB authigenic carbonates (Fig. 3, see also Table S5). Among isoprenoid hydrocarbons, the irregular tail-to-tail isoprenoids, such as crocetane, are often coeluted with phytane. Considering that the isotopic compositions of the crocetane/phytane peaks in CB carbonates were with − 28.5 to − 25.4‰ (Table S5), i.e., not ^13^C-depleted, it appears that crocetane was nearly absent in our samples, but phytane derived from the degradation of chlorophyll and other pigments was present with the values of 0.01–0.05 μg g^− 1^ dry weight (dw). The C_25_ compound 2,6,10,15,19-pentamethylicosane (PMI) was detected in the range of 0.02–0.04 μg g^− 1^ dw. Moreover, isoprenoid dialkyl glycerol diethers (isoprenoid DGDs), such as archaeol and *sn*-2-hydroxyarchaeol, were the most predominant archaeal lipids in the samples, displaying concentration ranges of 0.02–0.18 μg g^− 1^ dw and 0.02–0.32 μg g^− 1^ dw, respectively. For bacterial lipids, non-isoprenoid DGDs (i.e., If, IIa, and IId) with anteiso pentadecyl moieties or cyclopropyl groups attached at both the *sn*-1 and *sn*-2 positions were identified, ranging between 0.01 and 0.11 μg g^− 1^ dw. With respect to other DGDs (series I, II, and III), we could not fully determine their presence due to the detection limit. Among fatty acids (FAs) detected, saturated FAs (e.g., C16:0 and C18:0) were the most predominant, ranging between 0.04 and 0.62 μg g^− 1^ dw (Table S5). Other FAs (e.g., i-C15:0, *ai*-C15:0, C16:1ω7, C18:1ω9, C18:1ω7, C20:0, and C22:0) were approximately 2 to 10 times lower in concentration (0.01–0.23 μg g^− 1^ dw) compared with the predominant saturated FAs (Table S5). The δ^13^C values of PMI ranged from − 88.2 to − 61.5‰ (Fig. 3, see also Table S5). The δ^13^C values of archaeol and *sn*-2-hydroxyarchaeol varied between − 102.9 and − 82.9‰ and between − 108.0 and − 87.5‰, respectively (Fig. 3, see also Table S5). Finally, the δ^13^C values of non-isoprenoid DGDs ranged from − 88.6 to − 50.6‰ (Fig. 3, see also Table S5).

### Microbial compositions

**Figure 4:**
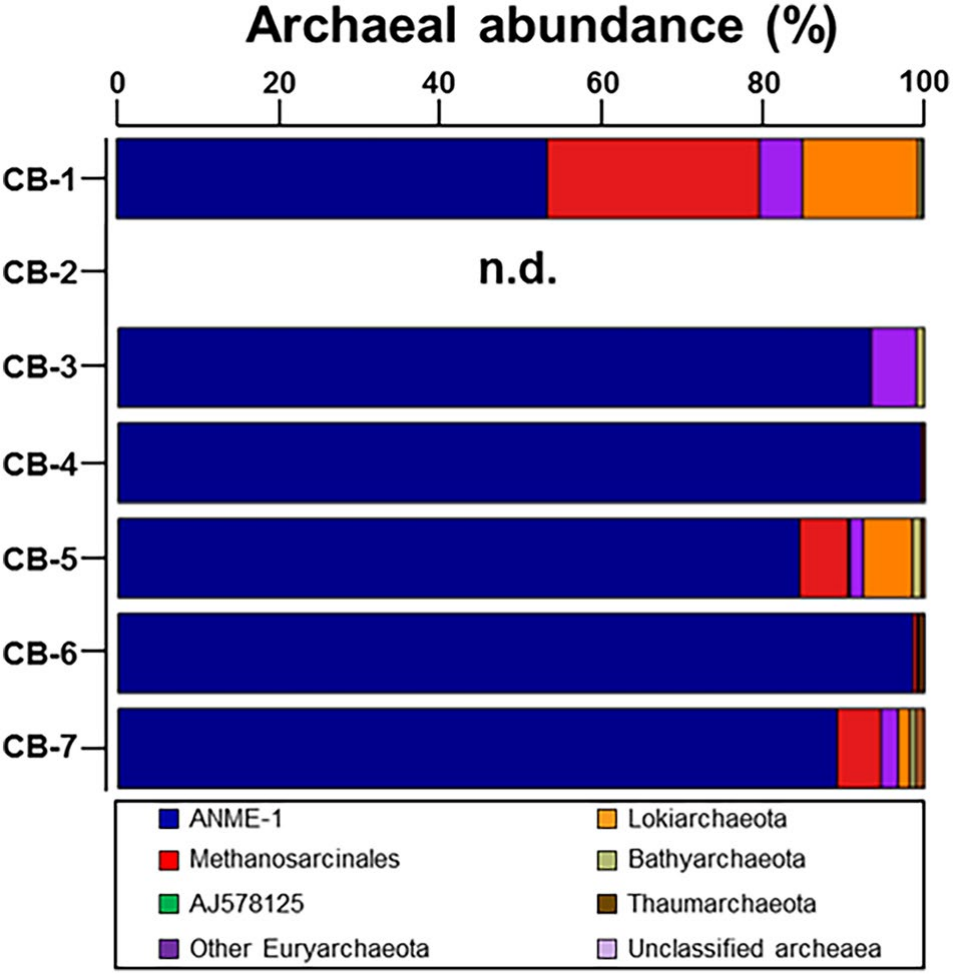
Relative abundances of archaea taxa at the phylum and order level of Euryarchaeota in authigenic carbonates of the Chukchi Borderland (CB). ‘n.d.’ denotes ‘not determined’.

The archaeal phyla detected in the studied samples were Euryarchaeota, Lokiarchaeota, Bathyarchaeota, and Thaumarchaeota (Table S6), with Euryarchaeota having the highest abundance, accounting for > 85% of the total archaeal sequences. Among them, the relative abundance of archaea ASV032, which belongs to ANME-1, was dominant (up to 99.5%; Fig. 4). Another archaea, ASV095, which belongs to *Methanosarcinales*, comprised 0.5% to 64.4% of archaeal sequences. In the bacterial communities, Firmicutes, Proteobacteria, Atribacteria, Actinobacteria, Bacteroidetes, Planctomycetes, and WM88 were predominant (Table S6).

## Discussion

### Constraints on fluid sources and environmental conditions during carbonate precipitation

Magnesium (Mg) calcite was the most dominant mineral (23–46 wt. %) in all carbonate samples retrieved from the CB mounds (Table 1). Generally, the environmental conditions during which carbonate precipitation occurs at cold seeps can be inferred from the mineralogical composition of carbonates^23,29^. For instance, aragonite precipitation is believed to be favored at sub-oxic conditions characterized by high dissolved sulfate concentrations in pore waters (i.e., near the seafloor), while high-Mg calcite and dolomite form preferentially under more reducing conditions and low sulfate levels^30^. Mg-bearing carbonate minerals such as dolomite are often encountered in relatively deeply buried sulfate-depleted sediment layers^31^. By analogy, the Mg-calcite carbonates encountered at the CB mounds most likely indicate precipitation under reducing conditions within the sediment column^32^. Moreover, considering that dissolved sulfide can catalyze dehydration of Mg^2+^ ions in fluids, enabling more rapid precipitation of Mg-calcites as opposed to aragonite^33^, the CB mounds may be subjected to anoxic conditions over long periods of time, since sulfide-enriched microenvironments could have been shaped by AOM processes under anoxic conditions. Furthermore, preferential precipitation of calcite over aragonite may occur at relatively low temperatures^34^, given that the temperature in the bottom seawaters of the Chukchi Sea is typically below 1 °C^21^. Thus, these properties could potentially explain the predominance of calcite over aragonite in the carbonate samples.

The measured δ^13^C_carb_ values (− 37.0 to − 32.8‰; Table 1 and Fig. S2) were significantly lower than the typical values (~ 0‰) of marine bicarbonate (i.e., dissolved inorganic carbon in seawater)^35^. In general, the δ^13^C_carb_ values reflect the source of dissolved carbon incorporated during carbonate precipitation^36^. The potential contributions of these different carbon isotopic signatures include (1) methane (δ^13^C_CH4_ for biogenic origin; < -60‰, and δ^13^C_CH4_ for thermogenic origin; − 50 to − 30‰) and (2) organic matter (ca. − 25‰)^37,38^. Furthermore, the extent of ^13^C-depletion in carbonates can be an indicator of specific microbial processes^39,40^. Considering that the carbon isotopic composition of ascending methane (δ^13^C_CH4_; − 95.7 to − 44.1‰) investigated at the CB mounds reflects a mixture of biogenic and thermogenic sources^22^, the microbial oxidation using this ascending methane can produce ^13^C-depleted bicarbonate (HCO_3_^−^) in sediments. We could not determine the precise age of carbonate samples using the U-Th method, due to the large amounts of detrital Th in our samples. Nonetheless, considering the sampling depths of our carbonate samples (see Fig. S1), they might be weakly related to the current sulfate-methane transition zone (SMTZ). Thus, the isotopic depletion of all carbonates (δ^13^C_carb_ < − 30‰) at the CB mounds suggests that a substantial fraction of the carbon involved in carbonate precipitation was derived from the ascending methane-rich fluids, likely related to ancient methane releases similar to those that occurred in the Barents Sea^41^. Pore fluids become ^18^O-enriched (δ^18^O_fluid_) during clay mineral dehydration or gas hydrate dissociation, which results in high δ^18^O_carb_ values^30,42,43^. At the studied site, gas hydrate layers have been identified at sediment depths below 3–4 m^22^. In this regard, the calcite-dominated carbonates (δ^18^O_carb_; 5.1 ± 0.6‰ VPDB (n = 7), Table 1 and Fig. S2) investigated at the CB mounds display ^18^O-enriched isotopic signatures. Therefore, these results may indicate that they precipitated from similarly enriched pore waters, resulting from gas hydrate dissociation.

### Trace element enrichments in authigenic carbonates

Compared to marine bioskeletal carbonates (i.e., JLs-1 limestone), most trace elements in the carbonate fractions are enriched in the studied cold seep carbonates, except for Ti, Ba, and W (Fig. 2a). These enrichments are well explained as reflecting the geochemical composition of surrounding pore waters, inherited from various early diagenetic processes that typically result in selective trace element enrichment^44^. In cold seeps, authigenic carbonates dominated by calcite typically incorporate higher amounts of trace elements (including REEs) compared to aragonite-rich samples, which form near the seafloor^27^. Moreover, sustained anoxic conditions in cold seeps generally explain why many redox-sensitive trace elements (Mo, U, Ni, V, Co, and Zn) are enriched in corresponding authigenic carbonates, such as in the CB carbonate samples^45^. Enrichment of Mo typically relates to Fe–Mn oxyhydroxide cycling in subsurface sediments and/or sulfide mineral formation, resulting from the presence of hydrogen sulfide in pore waters released from AOM^46^. Similarly, the observed enrichments identified for Ni, Co, and Zn reflect the remineralization of organic matter in sub-surface sediments, which also results in enriched sedimentary pore waters^47,48^.

The total lipid extracts of CB carbonates displayed particular trace element enrichments (Li, Ti, Ni, Co, Cu, Zn, and Mo) compared to the corresponding carbonate fractions (Fig. 2c). In contrast to the enrichments observed in leached carbonate fractions, which are directly inherited from the chemical composition of the pore waters from which they have precipitated, the enrichment associated with the lipid fractions hosted by authigenic carbonates can provide information on the bio-essential elements related to AOM-driven microbial activity at seeps^20^. Thus far, Ni and Zn have been identified in microbial organism cells, especially in seep-related microbial assemblages, where they are bound to specific sites of proteins and enzymes^49,50^. Co is present in cobamides involved in methyl group transfer during methanogenesis and methanotrophic processes^17^. Mo is bound to a pterin cofactor to form molybdopterin, which catalyzes electron redox reactions^50^, while Zn occurs as a single structural atom in both enzymes^50^ (e.g., methanol-coenzyme M methyltransferase enzyme (Mta) and Heterodisulfide reductase (Hdr)). Considering that microbial metalloenzymes play a key role in catalyzing major biogeochemical reactions, in particular AOM^51^, these enzymes may require some trace elements (e.g., Co, Ni, Mo, and Zn) as co-factors for electron transport or as catalytic centers at active sites^50^. Although trace elements (especially Cu and Ni) may be enriched in the organic (i.e., lipid) fractions due to their chemicophysical affinities to organic matter^45^, trace elements, such as Co, Ni, Mo, and Zn, were enriched, not only in the lipid fractions (Fig. 2b) but also in the carbonate fractions (Fig. 2a). Hence, the enrichment of Co, Ni, Mo, and Zn identified in the total lipid fractions associated with CB carbonates is most likely to reflect their implication in specific metal-rich enzymatic pathways related to AOM-driven methanotrophic activity. Fe is primarily present as Fe-S clusters used for electron transport and/or catalysis, while Ni is either bound to Fe-S clusters or in the center of a porphyrin unique to methanogen/methanotrophs containing cofactor F430^52^. Thus, their enrichment in the total lipid extracts separated from CB carbonates could similarly reflect their potential role as a limiting factor for methanotrophic activities because of their high bioavailability as essential metalloenzymes during AOM^53^.

Concerning Cu, which was also significantly enriched in the analyzed lipid fractions, it is worth mentioning that Cu-dependent anaerobic methanotrophic enzymes have not yet been identified, although Cu availability is important for aerobic methanotrophs^54^. For instance, an increase in Cu concentration can cause up to a 55-fold expression of particulate methane monooxygenase (pMMO) as a membrane protein found in aerobic methanotrophs^50^. Similar enrichments have been recently documented in cold seeps for light rare earth elements (e.g., La, Ce), also reflecting their preferential utility in aerobic methanotroph activity^55^. In the CB carbonates, the total lipid fractions extracted did not show any particular enrichment in light REE, indicating a decoupling between Cu and light REE. Given that Mg-calcite carbonates were predominantly precipitated under anoxic conditions in the CB mounds, the observed Cu enrichments in the CB carbonates would not be associated with the aerobic methanotrophy. Hence, additional studies are required to better assess the potential utility of Cu in AOM treatment. Similarly, it still needs to be elucidated why Li and Ti were enriched in the studied lipid fractions in the CB carbonates, and whether the potential bioavailability of Li and Ti as metalloenzymes could represent a limiting factor in methanogenesis and methanotrophic activity under anoxic conditions. Notably, these elements have previously been identified in microorganism cells, especially in seep-related microbial assemblages, where they were known to be bound to specific sites of proteins and enzymes^49,50^. Thus, to some degree, we speculate that the observed Cu, Li, and Ti enrichments in the total lipids of CB carbonates might be indicative of alternative microbial processes for AOM as metalloenzymes. Hence, in many physiological and biogeochemical aspects, further research is required to investigate the potential availability of Cu, Li, and Ti in anaerobic methanogens/methanotrophs in pure cultures.

### Microbial signatures in authigenic carbonates

In this study, irregular hydrocarbons (e.g., PMI) and isoprenoid DGDs (e.g., archaeol and *sn*-2-hydroxyarchaeol) were identified as AOM-related lipids, showing the predominance of isoprenoid DGDs in most CB carbonate samples (Fig. 3). The δ^13^C composition of these lipids indicated relatively ^13^C-depleted signatures, compared to those reported for methane in the CB mounds (Fig. 3). In general, methane-derived microbial lipids are depleted in ^13^C in comparison to the methane source as a result of isotopic fractionation during methane assimilation^56^. The more ^13^C-depleted isoprenoid DGDs (i.e., archaeol and *sn*-2-hydroxyarchaeol) may indicate the occurrence of methanotrophs involved in AOM. Notably, CB-6 showed the most predominant ^13^C-depleted isoprenoid DGDs. This ^13^C-depleted pattern could be closely linked to active AOM taking place in sediment layers where the SMTZ currently occurs^22^ (90–120 cm sediment depths). On the other hand, because the depth of the SMTZ in methane seeps can fluctuate as a result of the upward methane fluxes^57,58^, low amounts of ^13^C-depleted lipids investigated in other CB carbonate samples could alternatively be explained as a result of the diffusive migration of methane-rich fluids^59^. In this context, the low amount of ^13^C-depleted microbial lipids in the CB carbonates below and above the present SMTZ may potentially reflect the weak AOM activities as a fossil signature during the change in methane fluxes^60^. Non-isoprenoid DGDs (If, IIa, and IId) derived from thermophilic and halophilic SRB^61,62^ were also detected in the studied CB carbonates (Fig. 3). Overall, the abundance of bacterial lipids in the CB carbonates was lower than that of AOM-related archaeal lipids. Considering previous studies in other cold seepages, such as the North Sea^63^ and the Eastern Mediterranean Sea^64^, the observed pattern between both lipid groups appear to reflect the process of AOM-coupled sulfate reduction. Furthermore, these bacterial lipids are more enriched in ^13^C compared to archaeal lipids (∆^13^C = 10–15‰), which reflects their involvement in AOM as syntrophic partners^7,65^. Based on these isotopic fractionations, the ^13^C-depleted non-isoprenoid DGDs in the CB carbonates (CB-1, -2, -6, and -7) may serve as evidence of syntrophic organisms involved in AOM. However, the δ^13^C values of the non-isoprenoid DGDs (− 53.9 ± 3.6‰, n = 5) in other CB carbonates (CB-3, -4, and -5) were slightly enriched in ^13^C relative to the ascending methane reported in the CB mounds. Therefore, these ^13^C-enriched lipids may indicate that these compounds originate from a mixed source mediating AOM as well as other processes. Our results should be further confirmed by determining quantitative and qualitative properties of source organisms obtained from different analytical approaches, such as potassium hydroxide (KOH) hydrolysis.

Based on the microbial lipids and nucleic acids, the chemotaxonomy of key microbes involved in AOM can be determined^66^. Three groups of ANME (ANME-1, ANME-2, and ANME-3) have often been reported in various cold seep environments, which are related to methanogens from the order *Methanosarcinales* and *Methanomicrobiales*^58,67^. The microbial communities dominated by ANME-1 biosynthesize archaeol and PMI with relatively low amounts of pentamethylicosenes (PMEs), whereas ANME-2 is characterized by the predominance of *sn*-2-hydroxyarchaeol (relative to archaeol) and abundant PMEs and crocetane/crocetenes^66^. Together with the presence of PMI in CB carbonates, the ratio of *sn*-2-hydroxyarchaeol to archaeol varied within the range 0.9–1.8 (Fig. 3), suggesting the dominant occurrence of ANME-1, which lived under anaerobically sulfate-depleted conditions^68^. These distinctive lipid patterns in most CB carbonates were also supported by higher proportions of ASV032, which belongs to ANME-1 (Fig. 4). Furthermore, the co-occurrence of syntrophic bacterial partners associated with ANME-1 can be indirectly inferred from the abundance of bacterial lipids, such as non-isoprenoid DGDs and fatty acids^66^. To date, ^13^C-depleted non-isoprenoid DGDs have been mainly found in prominent ANME-1 systems^66^. Thus, the less ^13^C-depleted non-isoprenoid DGDs in the CB carbonates appear to be associated with an unknown SRB linked to AOM^69^. Furthermore, the low abundance of branched fatty acids (i.e., i-C15:0 and *ai*-C15:0) in the CB carbonates may also indicate a negligible presence of typical SRBs (i.e., *Desulfosarcina/Desulfococcus*) associated with ANME-1. This inference may be partially supported by the incomplete sequence of *Deltaproteobacteria*. Hence, these lipid characteristics in CB carbonates, in combination with 16S rRNA gene pyrosequencing data, suggest that SRBs, not belonging to *Deltaproteobacteria* but possibly to other bacterial communities, would have been involved in AOM in the CB mounds. Notably, ANME-1 has been microscopically shown to occur as a single cell in cold seep environments^70^. Accordingly, ANME-1-related syntrophic relationships within the CB mounds appear to be more linked to other bacterial communities than typical SRB consortia.

### Potential metalloenzymes involved in methanotrophic activity

**Figure 5:**
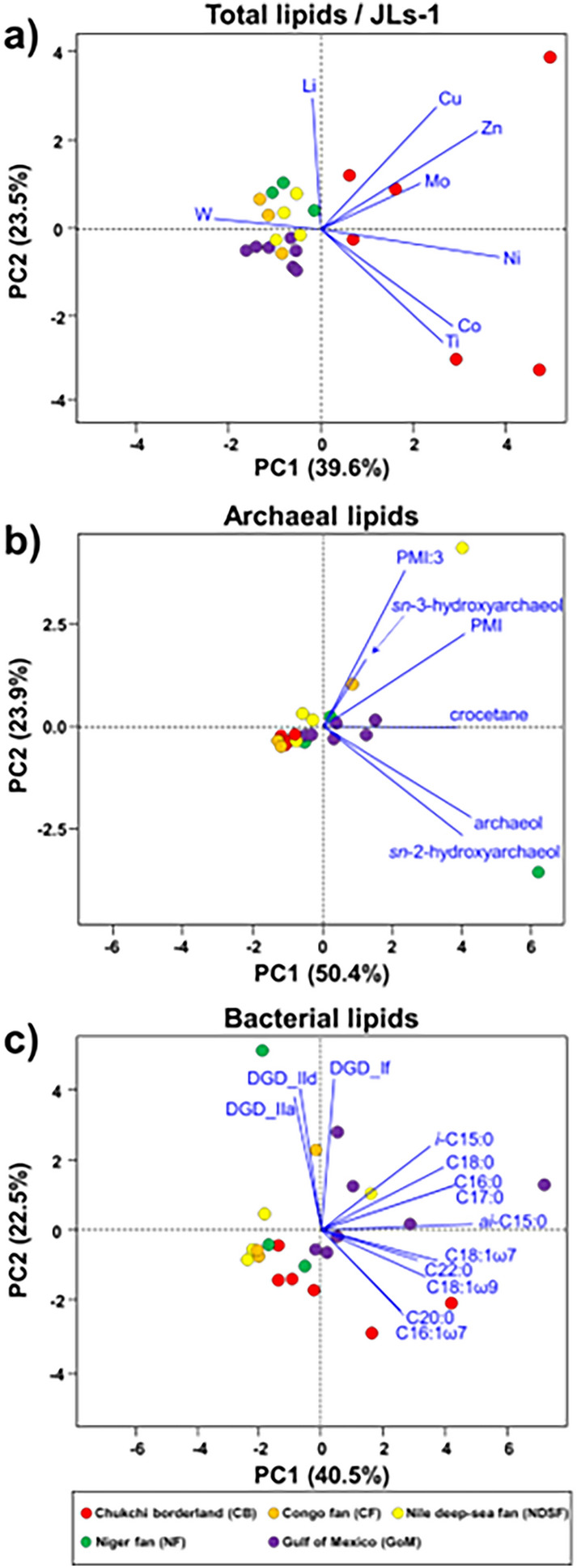
PCA results for (**a**) the trace metal data for the total lipids normalized to the values of the JLs-1 carbonate reference material, and for the concentration data of (**b**) archaeal lipids and (**c**) bacterial lipids.

**Figure 6:**
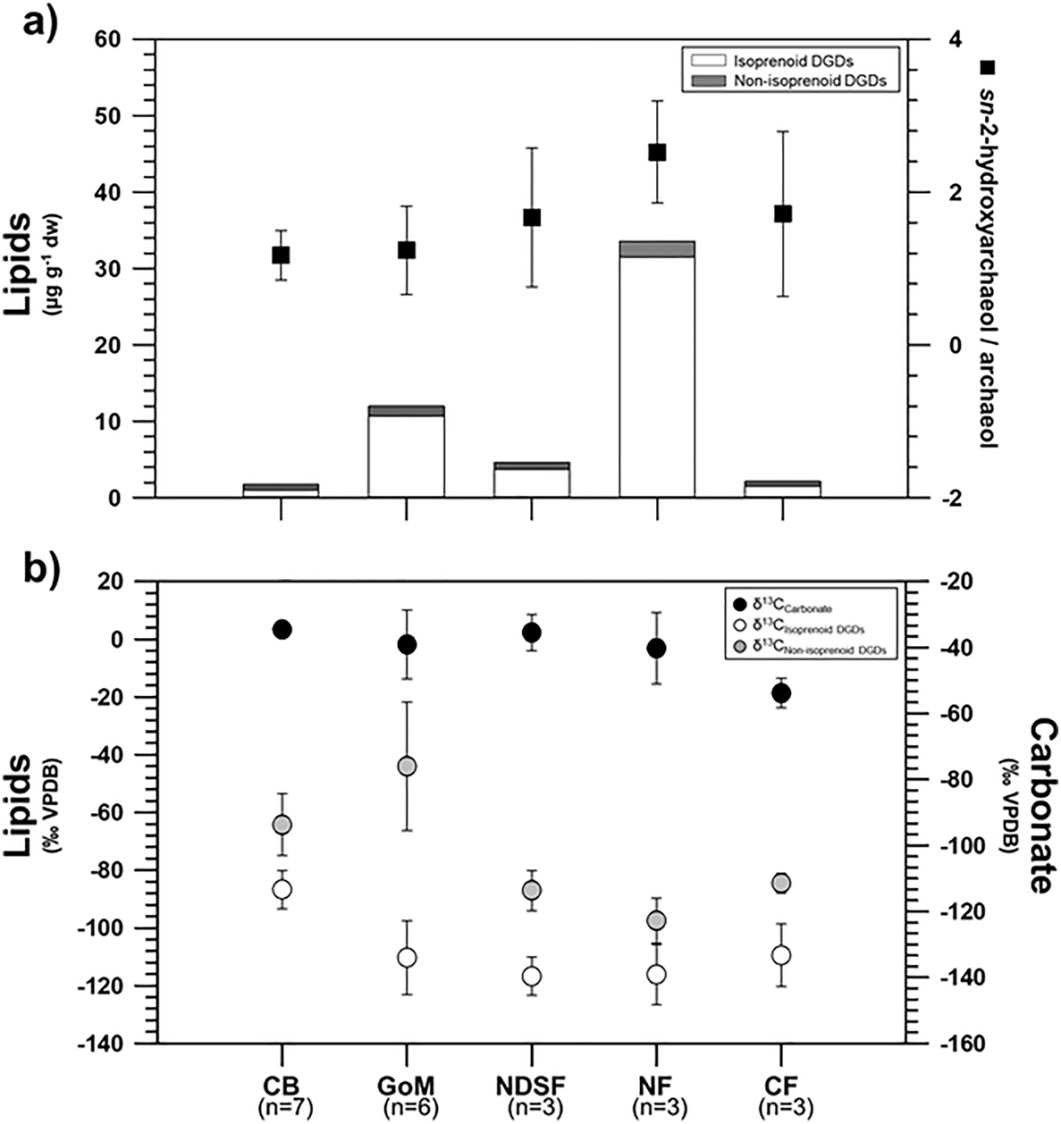
Comparison of lipid biomarker signatures of authigenic carbonates from the CB, Congo fan (CF), Nile deep-sea fan (NDSF), Niger fan (NF), and Gulf of Mexico (GoM): (**a**) concentrations of major lipid biomarkers and ratios of *sn*-2-hydroxyarchaeol relative to archaeol, and (**b**) carbon isotopic compositions of major lipid biomarkers with those of carbonates.

**Figure 7:**
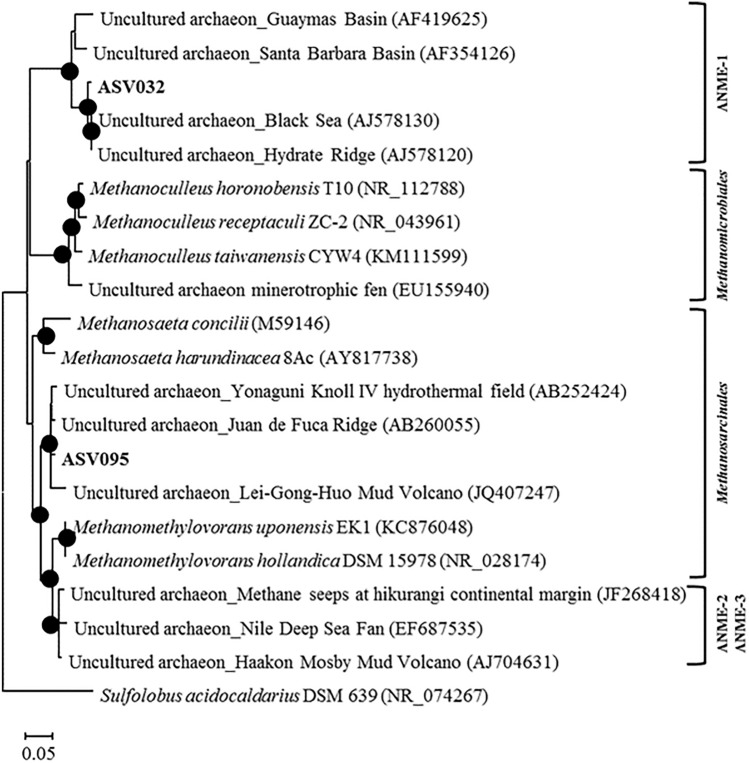
Phylogenetic tree of archaeal rDNA sequences obtained from authigenic carbonates.

To further investigate the distribution patterns of trace elements (W, Li, Ti, Ni, Co, Cu, Zn, and Mo) presumably acting as potential metalloenzymes involved in AOM at cold seeps, principal component analysis (PCA) was performed using the data for total lipid extracts normalized to JLs-1 (Fig. 5a). We used these data to compare authigenic carbonate samples collected from different regions worldwide instead of those normalized to associated carbonate fractions (Fig. S5). Although W was not detected in our total lipid samples of the CB, this element is included because pronounced lipid-bound W anomalies are reported at other seep sites worldwide^20^. PCA was also performed using the archaeal and bacterial lipid data to compare the CB signatures with those from other cold seeps^20^ (Fig. 5b–c). The PCA results show that the combined distribution of trace elements and bacterial lipids in CB carbonates differs from those reported for other methane seeps worldwide (e.g., Congo fan; CF, Nile deep-sea fan; NDSF, Niger fan; NF, and Gulf of Mexico; GoM). This finding suggests that the enrichment of Ni, Co, Zn, Cu, Ti, and Mo in carbonate-hosted lipid fractions could potentially be related to different methanotrophic communities and their metalloenzymes. This hypothesis is plausible because ANME-1 is predominant in CB carbonates, while ANME-1 and *Methanosarcinales* (potential ANME-2) co-exist at other sites^20^ (Figs. 6, 7). Ni-containing methyl-coenzyme MCR is the key enzyme in AOM^19^. Two different MCR homologues, designated as proteins I and II^71,72^ have been isolated from cold seep areas: protein I contains (17^2^S)-17^2^-methylthio-F430, whereas protein II contains F430^18,73^. Considering that protein I is predominantly detected in ANME-1^74^, we may infer that particular environmental conditions (e.g., temperature and partial pressure of methane) in the CB mound area specifically promote the synthesis of protein I for AOM^75^ compared to what can be observed at other seep sites. Future studies targeting deep gas-fluid sources and subsurface lithology are needed to unravel the crucial factors for determining discriminative ANME activities involved in the stimulated response under permafrost dissociation. The impact of lipid/enzyme degradation on the correlations between AOM-related lipids and trace elements should also be tested by comparing their relationships using data from modern and ancient carbonate samples. Previous studies have shown that there is a significant trace element enrichment difference between intra- and inter-crystalline lipid extractions in seep carbonates^48^. Hence, future studies should also explore the effect of the two-step extraction procedure on trace element distributions.

## Conclusions

We analyzed a series of authigenic carbonate samples collected at methane seeps from the southwestern slope of the CB using various inorganic and organic proxies of fluid sources and biogeochemical processes (stable isotopes, mineralogy, trace elements, lipid biomarkers, and nucleic acids). The absence of crocetane, the abundance of PMI, and the relatively low ratio of *sn*-2-hydroxyarchaeol to archaeol in the CB carbonates indicated the dominant occurrence of ANME-1, as confirmed by 16S rRNA gene pyrosequencing. Specific trace elements (Ni, Co, Zn, Cu, Ti, and Mo) appeared to be enriched in the total lipid fractions extracted from CB carbonates, confirming recent findings of their utility as metalloenzymes in microbial processes related to AOM at cold seeps. The patterns of trace element enrichment and microbial lipid assemblages in CB carbonates reveal distinct specific characteristics compared to those from other seeps worldwide (e.g., Congo fan, Nile deep-sea fan, Niger fan, and Gulf of Mexico). This finding suggests that trace element enrichment in carbonate-hosted lipid fractions at cold seeps could vary depending on the type of AOM microbial assemblages among the ANME groups. Further work is required to elucidate the role of trace elements, such as Co, Zn, Cu, Ti, and Mo, as potential metalloenzymes in AOM during N_2_ fixation or aerobic methane oxidation coupled with denitrification. In this regard, pure-culture experiments are necessary to understand their bioavailability for metalloenzymes involved in AOM.

## Material and methods

### Sampling

Previous geophysical surveys (e.g., multibeam and sub-bottom profiling) conducted at the southwestern slope of the CB^21^ led to the discovery of gas hydrate mound structures characterized by acoustic blanking below the crest at certain depth intervals (Fig. 1). During the ARA09C expedition in 2018, sediment cores (ARA09C-ST07, -ST14, and -ST04/06) were retrieved from three mound structures, together with various authigenic carbonate samples recovered at different sediment depths between 38 and 415 cm (Fig. 1). The recovered carbonate samples were cleaned with ultrapure Milli-Q (MQ) water and air-dried prior to being crushed into powder using an agate and mortar.

### Mineral and stable isotope analysis

The bulk authigenic carbonate samples were ground to a fine powder (< 20 µm particle size) for mineral analysis using a Philips X-ray diffraction (XRD) system at the laboratories of the Crystallography research group (Central Laboratory for Crystallography and Applied Material Sciences, University of Bremen). The X-ray source was a Cu anode operated at 40 kV and 45 mA using CuKα radiation equipped with a diffracted beam graphite monochromator. Step scans were run from 5 to 65° (2*θ)* with a step size of 0.02° and a count time of 2 s per step (0.6°/min). Mineral identification was performed using the Philips X’ Pert HighScore software, which provides a semi-quantitative value for each identified mineral on the basis of relative intensity ratio values. The oxygen and carbon isotopic compositions (δ^18^O and δ^13^C) of the carbonate samples were measured at Beta Analytic (service sample number: 536510 to 536,516). In brief, the sample was placed into a clean vial and dissolved in H_3_PO_4_ to release CO_2_ into the headspace of the vial. The sample was placed in a temperature-controlled tray (72 °C), where CO_2_ equilibration took place for one hour. Finally, helium (He) was added to the sample and a mixture of He and CO_2_ was injected into a Finnegan MAT 251 mass spectrometer. All isotopic values were expressed using the δ-notation relative to the Vienna Pee Dee Belemnite (VPDB) standard and were reported in per mil (‰) with a standard deviation of less than 0.3‰ for both δ^18^O and δ^13^C values.

### Trace element analysis

Homogenized carbonate samples (~ 5 g) were treated using the sequential extraction procedure previously described^20^ to selectively separate carbonate, sulfide, and detrital silicate fractions. We used the lipid fractions obtained from the solvent extraction method using a mixture of organic solvents (dichloromethane (DCM):methanol (MeOH), 2:1 v/v) to compare trace elements in lipid fractions with lipid biomarker data. The total lipid extracts (TLEs) were split into two aliquots for trace element analysis at IFREMER (Institut Français de Recherche pour l'Exploitation de la Mer; i.e., “French Research Institute for Exploitation of the Sea”) and lipid biomarker analysis at the Korea Polar Research Institute (KOPRI). All analyses were performed using an Element XR™ inductively coupled plasma-mass spectrometer (ICP-MS) at the Pôle Spectrométrie Océan (Brest, France). Polyatomic oxide and hydroxide interferences on the rare earth element (REE) were corrected using oxide formation rates determined by analyzing solutions of MQ–H_2_O, Ba + Ce, Pr + Nd, and Sm + Tb at the beginning of each measurement session and applied to all samples. Elemental concentrations were calculated using the Tm addition method^76,77^. The internal precision of all measurements was generally better than 5%. Repeated analyses of the JLs-1 reference material (Triassic limestone) were also performed, with a precision of < 10% for most trace elements, except for Li (11.8% relative standard deviation; RSD), Ti (61% RSD), and Zr (17% RSD). Note that the relatively poor RSD determined for Ti mostly reflects the very low abundance of this element in JLs-1 (4.1 µg/g). Significant Ti enrichments were measured in the lipid fractions, up to three orders of magnitude (Supplementary Tables); hence, much higher than the RSD determined for JLs-1. Therefore, we are confident that the measured Ti enrichments in the studied lipid extracts are meaningful, and thus, Ti data are still reported in this study. Because of the high Ba/REE ratios, several carbonate samples analyzed (including the carbonate reference material JLs-1) displayed anomalously high Eu (and to a lesser extent Gd) concentrations as a result of under-corrected interferences; hence, these two elements were not reported here.

### Lipid biomarker analysis

Detailed procedures for lipid biomarker analyses have been previously described^60^. In short, one-half of the TLE was dried over anhydrous Na_2_SO_4_ and treated with tetrabutylammonium sulfite reagent to remove elemental sulfur. The TLE was chromatographically separated into apolar and polar fractions over an Al_2_O_3_ column (activated for 2 h at 150 °C). The apolar fraction was eluted using hexane:DCM (9:1), and 40 µL of 5α-androstane (10 µg mL^− 1^) was added as an internal standard. The polar fraction was recovered with DCM:MeOH (1:1 v/v) as an eluent and divided into two aliquots, to which either C_22_ 7,16-diol (10 µg mL^− 1^) or C_19_ nonadecanoic acid (10 µg mL^− 1^) was added as an internal standard. Each aliquot was derivatized through silylation and methylation, prior to quantification by gas chromatography (GC) and identification by gas chromatography-mass spectrometry (GC–MS). GC and GC–MS conditions were as previously described^60^. Molecular compounds were determined by comparing their mass spectral fragmentation patterns and retention times with previously published data^60^. The δ^13^C values of lipid compounds were determined using an isotope ratio mass spectrometer (IRMS) connected to a GC via a combustion interface (glass tube packed with copper oxide, CuO, operated at 850 °C), as previously described^60^. Isotopic values were expressed as δ^13^C values per mil relative to VPDB. The δ^13^C values were corrected for the introduction of additional carbon atoms during silylation and methylation. The analytical errors were less than ± 0.4‰ for all lipid compounds.

### Nucleic acid analysis

Genomic DNA was extracted using the FastDNA Spin Kit for Soil (MP Biomedicals, USA). DNA samples were subjected to polymerase chain reaction (PCR) amplification, library preparation, and paired-end Illumina MiSeq sequencing (2 × 300 bp) at the Integrated Microbiome Resource (IMR), Dalhousie University, Canada (http://cgeb-imr.ca). The primer pairs 515F/ 926R and 956F/ 1401R targeting the V4–V5 and V6–V8 regions were used to amplify bacterial and archaeal 16S rRNA genes, respectively^78,79^. Amplicons were sequenced using the paired-end (2 × 300 bp) Illumina MiSeq system (Illumina, USA) at the Integrated Microbiome Resource (IMR), Dalhousie University, Canada (http://cgeb-imr.ca). Samples that were not amplified were excluded from further analyses. The adapter and primer sequences were removed using Cutadapt version 2.10^80^, and the resultant sequences were processed using DADA2 version 0.9.5^81^ to infer amplicon sequence variants (ASVs). For the quality trimming process, we applied a filtering option of maxN = 0, maxEE = c (2,2), and truncQ = 2. The low-quality tails of both reads were removed with truncLen = c (270,210). Denoising was performed after trimming based on the DADA2 error model. Sequences were dereplicated, and the core sample inference algorithm was applied to the dereplicated data. Paired reads were merged, and the chimeric sequences were removed. The following processes were performed after constructing a sequence table of ASVs to assign taxonomy using the software mothur^82^. Taxonomic assignments of representative ASV sequences were determined against the EzBiocloud database using sequence similarity searches^83^. After taxonomic assignment, archaea and unknown ASVs were removed for bacterial analysis, and bacteria and unknown ASVs were removed for archaeal analysis. All sequence data used in this study were deposited in the Sequence Read Archive at the National Center for Biotechnology Information under accession number PRJNA825649. Phylogenetic trees of major archaeal ASVs of Methanomicrobia with relative abundances greater than 3% were constructed using the maximum-likelihood algorithm^84^ with the general time reversible evolutionary model using MEGA X^85^. The robustness of the tree topologies was assessed using bootstrap analyses based on 1,000 replications of the sequences.

### Statistical analysis

To provide a general view of the variability of trace element abundances and microbial lipid distributions in the carbonate samples, gaps in the data set were first filled as previously described^86^. When some components were not determined, a value of one-half of the minimum value detected for that variable in the whole data set was set as the limit of detection. These values were then replaced by a random number between zero and the limit of detection. Finally, the fractional abundances of trace elements and microbial lipids were obtained by normalizing each concentration to the summed concentrations of all trace elements and microbial lipids, respectively. Based on the fractional abundances, PCA was performed using R software version 3.4.2 (package information; FactoMineR, Vienna, Austria).

## Supplementary Information

Supplementary Information.

## Data Availability

All DNA sequence data generated for this study were deposited in the Sequence Read Archive at the National Center for Biotechnology Information under accession number PRJNA825649.
