# Supplementary material for: Metalloenzyme signatures in authigenic carbonates from the Chukchi Borderlands in the western Arctic Ocean
Source: Sci Rep. 2022 Oct 5;12:16597. doi: 10.1038/s41598-022-21184-6 (PMC9534989; doi:10.1038/s41598-022-21184-6)
Supplement: Supplementary file 1 — Supplementary Information. [file 41598_2022_21184_MOESM1_ESM.docx]

**Supplementary Information**

**Metalloenzyme signatures in authigenic carbonates from the Chukchi Borderlands in the western Arctic Ocean**

Dong-Hun Lee^1,†^, Jung-Hyun Kim^2,*^, Yung Mi Lee^2^, Germain Bayon^3^, Dahae Kim^2^, Young Jin Joe^2^, Xudong Wang^4^, Kyung-Hoon Shin^1^, Young Keun Jin^2^

^1^Department of Marine Sciences and Convergent Technology, Hanyang University ERICA Campus, Ansan, 15588, Republic of Korea

^2^Korea Polar Research Institute, Incheon, 21990, Republic of Korea

^3^University Brest, CNRS, Ifremer, Geo-Ocean, F-29280 Plouzané, France

^4^Shanghai Engineering Research Center of Hadal Science and Technology, College of Marine Sciences, Shanghai Ocean University, Shanghai 201306, China

***Correspondence:**

Jung-Hyun Kim
jhkim123@kopri.re.kr

^†^Present address:

Marine Environment Research, Division, National Institute of Fisheries Science, Busan, South Korea

Table S1. Elemental concentrations for carbonate fractions (1M AA leachates) of authigenic carbonates (mg/kg) from the Chukchi Borderlands.

| **Sites** | **Sample ID** | Carbonate fraction (1M AA leachates) (ppm) | | | | | | | |  | |  | |  | |  | |  |  | |  | |  | |  | |  | |  | |  | |  | |  |  | |  | |  | |  | |  | |  |  | |  | |  | |  |  | Reference |
| --- | --- | --- | --- | --- | --- | --- | --- | --- | --- | --- | --- | --- | --- | --- | --- | --- | --- | --- | --- | --- | --- | --- | --- | --- | --- | --- | --- | --- | --- | --- | --- | --- | --- | --- | --- | --- | --- | --- | --- | --- | --- | --- | --- | --- | --- | --- | --- | --- | --- | --- | --- | --- | --- | --- | --- |
|  |  | Li | Ti | V | Mn | Co | Ni | Cu | Zn | | Rb | | Sr | | Zr | | Mo | | | Ba | | La | | Ce | | Pr | | Nd | | Sm | | Tb | | Dy | | | Y | | Ho | | Er | | Yb | | Lu | | | W | | Pb | | Th | | U |  |
| **Chukchi borderland**  **(CB)** | CB-1 | 11.66 | 0.53 | 8.07 | 118 | 2.03 | 3.18 | 0.09 | 6.72 | | 5.91 | | 3156 | | 0.34 | | 0.25 | | | 516 | | 2.84 | | 6.26 | | 0.79 | | 3.98 | | 1.04 | | 0.17 | | 0.93 | | | 6.26 | | 0.19 | | 0.49 | | 0.38 | | 0.06 | | | 0.01 | | 0.24 | | 0.04 | | 1.54 | This study |
|  | CB-2 | 4.26 | 3.77 | 14.12 | 1268 | 0.91 | 2.83 | 0.18 | 9.52 | | 1.78 | | 1722 | | 0.70 | | 10.56 | | | 32 | | 4.94 | | 11.40 | | 1.26 | | 5.85 | | 1.51 | | 0.30 | | 1.92 | | | 23.57 | | 0.46 | | 1.40 | | 1.38 | | 0.24 | | | 0.01 | | 0.41 | | 0.61 | | 83.62 | This study |
|  | CB-3 | 4.52 | 3.23 | 6.13 | 1641 | 1.21 | n.d. | 0.49 | 5.52 | | 1.93 | | 1177 | | 0.69 | | 1.06 | | | 30 | | 2.57 | | 6.80 | | 0.81 | | 3.66 | | 0.92 | | 0.13 | | 0.69 | | | 4.26 | | 0.13 | | 0.33 | | 0.27 | | 0.04 | | | 0.01 | | 0.65 | | 0.32 | | 1.06 | This study |
|  | CB-4 | 6.14 | 0.40 | 5.26 | 62 | 1.41 | 1.69 | 0.21 | 6.35 | | 1.91 | | 1692 | | 0.67 | | 0.10 | | | 272 | | 1.21 | | 3.04 | | 0.38 | | 1.80 | | 0.48 | | 0.08 | | 0.45 | | | 2.88 | | 0.09 | | 0.24 | | 0.20 | | 0.03 | | | 0.01 | | 0.28 | | 0.20 | | 0.34 | This study |
|  | CB-5 | 8.32 | 0.63 | 4.53 | 670 | 3.17 | 3.96 | 0.07 | 5.58 | | 3.72 | | 1912 | | 0.13 | | 4.85 | | | 60 | | 5.00 | | 11.65 | | 1.31 | | 6.21 | | 1.59 | | 0.30 | | 1.83 | | | 15.06 | | 0.40 | | 1.16 | | 1.05 | | 0.18 | | | 0.01 | | 0.32 | | 0.05 | | 4.79 | This study |
|  | CB-6 | 7.33 | 0.63 | 8.10 | 77 | 1.50 | 2.71 | 0.24 | 13.02 | | 2.46 | | 2392 | | 0.32 | | 0.23 | | | 476 | | 1.87 | | 4.54 | | 0.58 | | 2.72 | | 0.71 | | 0.13 | | 0.74 | | | 5.33 | | 0.15 | | 0.43 | | 0.37 | | 0.06 | | | 0.01 | | 0.51 | | 0.03 | | 1.42 | This study |
|  | CB-7 | 15.05 | 0.80 | 11.87 | 213 | 2.57 | 3.78 | 0.16 | 13.86 | | 5.73 | | 3659 | | 0.14 | | 0.33 | | | 788 | | 4.24 | | 9.66 | | 1.22 | | 6.65 | | 1.85 | | 0.39 | | 2.36 | | | 16.84 | | 0.51 | | 1.41 | | 1.12 | | 0.19 | | | 0.01 | | 0.39 | | 0.02 | | 9.95 | This study |
| **Congo Fan**  **(CF)** | CF-1 | 4.16 | 0.64 | 3.16 | 413 | 0.19 | 1.14 | 0.12 | 4.42 | | 0.64 | | 1535 | | 0.10 | | 0.71 | | | 798 | | 2.23 | | 8.21 | | 0.70 | | 2.92 | | 0.64 | | 0.10 | | 0.59 | | | 4.28 | | 0.13 | | 0.40 | | 0.38 | | 0.06 | | | 0.02 | | 0.50 | | 0.37 | | 8.07 | Wang et al., 2019 |
|  | CF-2 | 2.86 | 0.60 | 7.48 | 76 | 0.19 | 0.75 | 0.04 | 1.83 | | 0.34 | | 1121 | | 0.11 | | 0.41 | | | 330 | | 3.24 | | 8.50 | | 0.89 | | 3.80 | | 0.82 | | 0.14 | | 0.87 | | | 6.43 | | 0.20 | | 0.59 | | 0.52 | | 0.08 | | | 0.01 | | 0.11 | | 0.42 | | 7.50 | Wang et al., 2019 |
|  | CF-3 | 3.22 | 0.56 | 1.63 | 70 | 0.75 | 3.00 | 0.19 | 2.86 | | 0.32 | | 10409 | | 0.13 | | 0.07 | | | 10 | | 5.65 | | 11.69 | | 1.37 | | 5.53 | | 0.93 | | 0.10 | | 0.53 | | | 3.51 | | 0.11 | | 0.28 | | 0.22 | | 0.03 | | | 0.02 | | 0.08 | | 0.47 | | 6.23 | Wang et al., 2019 |
| **Nile Deep-Sea Fan**  **(NDSF)** | NDSF-1 | 1.29 | 0.31 | 8.79 | 266 | 0.54 | 1.90 | 0.24 | 3.87 | | 0.76 | | 9753 | | 0.77 | | 0.18 | | | 18 | | 3.10 | | 6.80 | | 0.77 | | 3.29 | | 0.69 | | 0.11 | | 0.64 | | | 4.06 | | 0.13 | | 0.36 | | 0.31 | | 0.05 | | | 0.01 | | 0.11 | | 0.31 | | 7.02 | Wang et al., 2019 |
|  | NDSF-2 | 3.31 | 1.27 | 1.77 | 117 | 2.42 | 3.82 | 1.92 | 3.94 | | 0.57 | | 1281 | | 0.41 | | 0.06 | | | 30 | | 8.41 | | 16.56 | | 2.06 | | 8.91 | | 2.00 | | 0.33 | | 1.98 | | | 13.18 | | 0.41 | | 1.12 | | 0.92 | | 0.14 | | | 0.01 | | 0.81 | | 0.40 | | 7.50 | Wang et al., 2019 |
|  | NDSF-3 | 2.10 | 0.97 | 3.68 | 64 | 0.29 | 1.59 | 3.34 | 5.07 | | 0.50 | | 7619 | | 0.41 | | 0.05 | | | 152 | | 4.44 | | 6.85 | | 1.07 | | 4.59 | | 0.99 | | 0.17 | | 1.06 | | | 9.06 | | 0.23 | | 0.65 | | 0.53 | | 0.08 | | | 0.01 | | 1.01 | | 0.36 | | 3.93 | Wang et al., 2019 |
|  | NDSF-4 | 1.39 | 0.92 | 3.30 | 115 | 0.38 | 0.93 | 1.96 | 2.84 | | 0.37 | | 9657 | | 0.58 | | 0.06 | | | 444 | | 2.63 | | 4.38 | | 0.66 | | 2.94 | | 0.65 | | 0.11 | | 0.67 | | | 5.58 | | 0.15 | | 0.41 | | 0.33 | | 0.05 | | | 0.01 | | 0.49 | | 0.20 | | 4.58 | Wang et al., 2019 |
| **Niger Fan**  **(NF)** | NF-1 | 0.62 | 0.30 | 4.67 | 4 | 0.03 | 0.72 | 0.21 | 1.53 | | 0.12 | | 10563 | | 0.02 | | 5.13 | | | 32 | | 0.10 | | 0.24 | | 0.02 | | 0.10 | | 0.02 | | 0.00 | | 0.02 | | | 0.20 | | 0.00 | | 0.01 | | 0.01 | | 0.00 | | | 0.02 | | 0.13 | | 0.01 | | 3.95 | Wang et al., 2019 |
|  | NF-2 | 1.12 | 0.36 | 6.25 | 20 | 0.22 | 0.63 | 0.13 | 1.50 | | 0.30 | | 10327 | | 0.08 | | 0.71 | | | 17 | | 4.36 | | 9.48 | | 0.91 | | 3.73 | | 0.55 | | 0.04 | | 0.21 | | | 1.25 | | 0.04 | | 0.09 | | 0.07 | | 0.01 | | | 0.01 | | 0.40 | | 0.19 | | 6.88 | Wang et al., 2019 |
|  | NF-3 | 3.01 | 0.53 | 14.46 | 419 | 0.33 | 1.05 | 0.03 | 6.12 | | 1.11 | | 395 | | 0.47 | | 0.19 | | | 65 | | 7.70 | | 24.62 | | 2.33 | | 10.30 | | 2.32 | | 0.41 | | 2.61 | | | 18.20 | | 0.59 | | 1.78 | | 1.67 | | 0.25 | | | 0.01 | | 0.32 | | 1.20 | | 7.77 | Wang et al., 2019 |
|  | NF-4 | 4.90 | 0.13 | 0.69 | 880 | 1.14 | 6.28 | 0.80 | 11.76 | | 1.71 | | 485 | | 0.14 | | 0.07 | | | 100 | | 15.97 | | 39.61 | | 4.25 | | 18.13 | | 3.73 | | 0.53 | | 3.07 | | | 21.00 | | 0.65 | | 1.86 | | 1.64 | | 0.25 | | | 0.01 | | 0.18 | | 2.31 | | 1.36 | Wang et al., 2019 |
| **Gulf of Mexico**  **(GoM)** | GoM-1 | 0.84 | 0.21 | 1.20 | 167 | 0.54 | 1.60 | 0.90 | 2.07 | | 0.92 | | 15455 | | 0.45 | | 0.07 | | | 92 | | 3.31 | | 4.88 | | 0.73 | | 3.06 | | 0.62 | | 0.08 | | 0.47 | | | 3.12 | | 0.10 | | 0.25 | | 0.20 | | 0.03 | | | 0.01 | | 0.11 | | 0.21 | | 8.21 | Wang et al., 2019 |
|  | GoM-2 | 2.12 | 0.14 | 1.64 | 295 | 0.52 | 1.25 | 0.46 | 3.68 | | 0.71 | | 1074 | | 0.44 | | 0.12 | | | 162 | | 1.84 | | 3.17 | | 0.41 | | 1.74 | | 0.38 | | 0.06 | | 0.37 | | | 3.00 | | 0.08 | | 0.23 | | 0.21 | | 0.03 | | | 0.01 | | 0.03 | | 0.07 | | 1.19 | Wang et al., 2019 |
|  | GoM-3 | 0.83 | 0.06 | 0.83 | 91 | 0.08 | 4.32 | 1.68 | 2.64 | | 0.47 | | 12430 | | 0.34 | | 0.13 | | | 31 | | 3.83 | | 2.43 | | 0.85 | | 3.73 | | 0.66 | | 0.07 | | 0.38 | | | 2.32 | | 0.07 | | 0.19 | | 0.14 | | 0.02 | | | 0.01 | | 0.29 | | 0.08 | | 9.30 | Wang et al., 2019 |
|  | GoM-4 | 3.88 | 0.09 | 1.39 | 564 | 0.60 | 4.73 | 1.23 | 5.44 | | 0.74 | | 3051 | | 0.12 | | 0.09 | | | 652 | | 2.90 | | 3.60 | | 0.70 | | 3.20 | | 0.75 | | 0.12 | | 0.69 | | | 5.49 | | 0.15 | | 0.39 | | 0.31 | | 0.05 | | | 0.01 | | 0.06 | | 0.07 | | 9.13 | Wang et al., 2019 |
|  | GoM-5 | 2.27 | 0.26 | 2.21 | 160 | 0.51 | 1.67 | 0.85 | 3.35 | | 0.59 | | 1141 | | 0.34 | | 0.06 | | | 295 | | 3.33 | | 4.54 | | 0.77 | | 3.39 | | 0.75 | | 0.12 | | 0.73 | | | 5.62 | | 0.16 | | 0.43 | | 0.35 | | 0.05 | | | 0.01 | | 0.13 | | 0.09 | | 0.50 | Wang et al., 2019 |
|  | GoM-6 | 2.77 | 0.09 | 1.95 | 182 | 0.53 | 3.19 | 1.19 | 3.52 | | 0.71 | | 1445 | | 0.20 | | 0.06 | | | 318 | | 5.99 | | 5.92 | | 1.39 | | 6.26 | | 1.42 | | 0.24 | | 1.42 | | | 11.07 | | 0.31 | | 0.83 | | 0.64 | | 0.10 | | | 0.01 | | 0.22 | | 0.09 | | 0.50 | Wang et al., 2019 |
|  | GoM-7 | 2.14 | 0.94 | 6.90 | 167 | 0.42 | 1.18 | 0.85 | 3.86 | | 0.64 | | 1228 | | 1.08 | | 0.07 | | | 657 | | 4.94 | | 9.38 | | 1.11 | | 4.53 | | 0.95 | | 0.15 | | 0.88 | | | 6.20 | | 0.19 | | 0.52 | | 0.46 | | 0.07 | | | 0.01 | | 0.14 | | 0.44 | | 5.17 | Wang et al., 2019 |

n.d. indicates ‘not determined’.

Table S2. Elemental concentrations for sulfide fractions (3M HNO_3_ leachates) of authigenic carbonates (mg/kg) from the Chukchi Borderlands.

| **Sites** | **Sample ID** | Sulfide fraction (3M HNO_3_ leachates) (ppm) | | | | | | | |  | |  | |  | |  | |  | |  |  |  | |  | |  | |  | |  | |  |  |  | |  | |  | |  | |  |  | |  | |  | |  | |  |  | | Reference |
| --- | --- | --- | --- | --- | --- | --- | --- | --- | --- | --- | --- | --- | --- | --- | --- | --- | --- | --- | --- | --- | --- | --- | --- | --- | --- | --- | --- | --- | --- | --- | --- | --- | --- | --- | --- | --- | --- | --- | --- | --- | --- | --- | --- | --- | --- | --- | --- | --- | --- | --- | --- | --- | --- | --- |
|  |  | Li | Ti | V | Mn | Co | Ni | Cu | Zn | | Rb | | Sr | | Zr | | Mo | | Ba | | La | | Ce | | Pr | | Nd | | Sm | | Tb | | Dy | | Y | | Ho | | Er | | Yb | | | Lu | | W | | Pb | | Th | | | U |  |
| **Chukchi borderland**  **(CB)** | CB-1 | 2.79 | 13.12 | 4.32 | 52 | 1.59 | 2.80 | 2.54 | 11.03 | | 2.28 | | 212 | | 1.83 | | 0.17 | | 66 | | 1.33 | | 3.00 | | 0.40 | | 1.71 | | 0.42 | | 0.06 | | 0.34 | | 1.83 | | 0.06 | | 0.16 | | 0.13 | | | 0.02 | | 0.00 | | 1.93 | | 0.76 | | | 0.16 | This study |
|  | CB-2 | 2.55 | 12.66 | 5.90 | 267 | 1.28 | 2.16 | 2.81 | 8.41 | | 2.54 | | 509 | | 1.61 | | 2.81 | | 7 | | 1.82 | | 3.77 | | 0.48 | | 2.05 | | 0.50 | | 0.08 | | 0.52 | | 4.53 | | 0.11 | | 0.33 | | 0.32 | | | 0.05 | | 0.01 | | 2.03 | | 0.79 | | | 10.23 | This study |
|  | CB-3 | 2.53 | 15.88 | 4.00 | 352 | 1.79 | 2.69 | 3.83 | 9.67 | | 2.64 | | 206 | | 2.34 | | 0.28 | | 11 | | 2.17 | | 4.93 | | 0.61 | | 2.57 | | 0.60 | | 0.09 | | 0.50 | | 2.97 | | 0.10 | | 0.26 | | 0.22 | | | 0.03 | | 0.01 | | 2.41 | | 1.02 | | | 0.52 | This study |
|  | CB-4 | 2.34 | 10.43 | 3.95 | 80 | 1.60 | 2.83 | 4.30 | 11.31 | | 2.28 | | 217 | | 2.13 | | 0.08 | | 55 | | 1.41 | | 3.39 | | 0.47 | | 2.05 | | 0.52 | | 0.08 | | 0.42 | | 2.26 | | 0.08 | | 0.19 | | 0.14 | | | 0.02 | | 0.00 | | 2.38 | | 0.92 | | | 0.14 | This study |
|  | CB-5 | 2.50 | 13.79 | 5.37 | 397 | 2.19 | 2.91 | 2.27 | 8.68 | | 2.31 | | 210 | | 1.69 | | 0.62 | | 13 | | 2.01 | | 4.37 | | 0.54 | | 2.29 | | 0.55 | | 0.10 | | 0.58 | | 3.84 | | 0.12 | | 0.34 | | 0.33 | | | 0.05 | | 0.01 | | 1.79 | | 0.84 | | | 0.68 | This study |
|  | CB-6 | 3.29 | 14.01 | 6.14 | 48 | 1.36 | 2.64 | 3.78 | 11.86 | | 2.20 | | 257 | | 3.27 | | 0.14 | | 80 | | 2.22 | | 5.15 | | 0.65 | | 2.67 | | 0.61 | | 0.09 | | 0.49 | | 2.92 | | 0.09 | | 0.24 | | 0.19 | | | 0.03 | | 0.01 | | 2.48 | | 1.03 | | | 0.33 | This study |
|  | CB-7 | 2.37 | 16.30 | 3.94 | 100 | 1.72 | 3.05 | 3.52 | 10.32 | | 2.61 | | 271 | | 2.11 | | 0.16 | | 234 | | 2.03 | | 4.52 | | 0.59 | | 2.57 | | 0.65 | | 0.12 | | 0.69 | | 4.17 | | 0.14 | | 0.39 | | 0.36 | | | 0.05 | | 0.01 | | 2.19 | | 1.00 | | | 1.12 | This study |
| **Congo Fan**  **(CF)** | CF-1 | 1.28 | 9.51 | 1.85 | 95 | 0.37 | 1.94 | 2.05 | 3.81 | | 1.32 | | 276 | | 0.48 | | 0.36 | | 862 | | 1.00 | | 3.46 | | 0.30 | | 1.20 | | 0.25 | | 0.03 | | 0.20 | | 1.52 | | 0.04 | | 0.13 | | 0.13 | | | 0.02 | | 0.00 | | 0.56 | | 0.47 | | | 1.52 | Wang et al., 2019 |
|  | CF-2 | 1.18 | 11.78 | 4.18 | 38 | 0.47 | 1.61 | 2.06 | 3.51 | | 1.27 | | 276 | | 0.56 | | 0.44 | | 110 | | 1.70 | | 4.54 | | 0.46 | | 1.88 | | 0.39 | | 0.06 | | 0.39 | | 3.09 | | 0.09 | | 0.25 | | 0.24 | | | 0.03 | | 0.00 | | 0.58 | | 0.54 | | | 1.99 | Wang et al., 2019 |
|  | CF-3 | 1.72 | 19.23 | 9.27 | 20 | 0.58 | 2.16 | 1.13 | 5.13 | | 1.01 | | 1757 | | 0.66 | | 0.36 | | 4 | | 2.25 | | 5.21 | | 0.54 | | 2.06 | | 0.35 | | 0.04 | | 0.21 | | 1.48 | | 0.04 | | 0.11 | | 0.09 | | | 0.01 | | 0.01 | | 0.80 | | 0.71 | | | 1.55 | Wang et al., 2019 |
| **Nile Deep-Sea Fan**  **(NDSF)** | NDSF-1 | 0.70 | 15.11 | 10.92 | 108 | 1.87 | 3.38 | 5.07 | 4.98 | | 1.53 | | 1967 | | 4.01 | | 2.16 | | 5 | | 2.83 | | 6.67 | | 0.79 | | 3.21 | | 0.65 | | 0.09 | | 0.51 | | 3.13 | | 0.10 | | 0.26 | | 0.21 | | | 0.03 | | 0.00 | | 0.81 | | 0.51 | | | 1.73 | Wang et al., 2019 |
|  | NDSF-2 | 1.39 | 33.08 | 11.93 | 100 | 3.84 | 4.44 | 4.11 | 3.34 | | 1.21 | | 229 | | 3.72 | | 0.21 | | 10 | | 3.60 | | 8.80 | | 0.87 | | 3.58 | | 0.75 | | 0.12 | | 0.67 | | 5.09 | | 0.14 | | 0.38 | | 0.32 | | | 0.05 | | 0.01 | | 1.91 | | 0.44 | | | 1.90 | Wang et al., 2019 |
|  | NDSF-3 | 0.82 | 20.93 | 5.83 | 20 | 0.32 | 1.53 | 2.59 | 2.85 | | 0.85 | | 1534 | | 1.23 | | 0.05 | | 42 | | 1.57 | | 2.55 | | 0.38 | | 1.56 | | 0.32 | | 0.05 | | 0.30 | | 2.90 | | 0.06 | | 0.17 | | 0.15 | | | 0.02 | | 0.01 | | 0.54 | | 0.18 | | | 0.93 | Wang et al., 2019 |
|  | NDSF-4 | 0.79 | 14.75 | 4.31 | 39 | 0.39 | 1.54 | 2.40 | 2.57 | | 0.78 | | 1958 | | 1.63 | | 0.27 | | 1110 | | 1.43 | | 2.51 | | 0.35 | | 1.45 | | 0.30 | | 0.04 | | 0.26 | | 2.30 | | 0.05 | | 0.14 | | 0.12 | | | 0.02 | | 0.01 | | 0.57 | | 0.23 | | | 1.21 | Wang et al., 2019 |
| **Niger Fan**  **(NF)** | NF-1 | 0.23 | 2.01 | 1.15 | 1 | 0.03 | 0.29 | 0.32 | 0.41 | | 0.10 | | 2660 | | 0.02 | | 0.49 | | 5 | | 0.08 | | 0.16 | | 0.02 | | 0.06 | | 0.01 | | 0.00 | | 0.01 | | 0.10 | | 0.00 | | 0.01 | | 0.00 | | | 0.00 | | 0.01 | | 0.04 | | 0.01 | | | 1.54 | Wang et al., 2019 |
|  | NF-2 | 0.34 | 4.27 | 2.31 | 4 | 0.30 | 0.66 | 0.68 | 0.69 | | 0.25 | | 2302 | | 0.13 | | 0.31 | | 4 | | 1.31 | | 2.75 | | 0.26 | | 1.04 | | 0.16 | | 0.01 | | 0.06 | | 0.45 | | 0.01 | | 0.03 | | 0.02 | | | 0.00 | | 0.00 | | 0.15 | | 0.07 | | | 1.74 | Wang et al., 2019 |
|  | NF-3 | 0.98 | 11.50 | 9.29 | 138 | 0.83 | 1.89 | 1.81 | 2.93 | | 1.83 | | 74 | | 1.43 | | 1.41 | | 19 | | 3.65 | | 11.76 | | 1.10 | | 4.73 | | 1.04 | | 0.18 | | 1.17 | | 9.18 | | 0.26 | | 0.79 | | 0.76 | | | 0.11 | | 0.01 | | 0.64 | | 0.63 | | | 2.58 | Wang et al., 2019 |
|  | NF-4 | 1.96 | 10.86 | 37.56 | 445 | 2.30 | 7.34 | 2.91 | 12.32 | | 4.34 | | 54 | | 1.73 | | 0.12 | | 40 | | 7.73 | | 20.59 | | 2.06 | | 8.37 | | 1.69 | | 0.23 | | 1.34 | | 9.51 | | 0.28 | | 0.79 | | 0.74 | | | 0.11 | | 0.01 | | 1.96 | | 1.90 | | | 0.39 | Wang et al., 2019 |
| **Gulf of Mexico**  **(GoM)** | GoM-1 | 1.58 | 18.57 | 10.78 | 92 | 1.26 | 2.44 | 3.13 | 5.24 | | 2.14 | | 2193 | | 2.23 | | 0.34 | | 20 | | 2.14 | | 3.96 | | 0.51 | | 2.02 | | 0.41 | | 0.06 | | 0.29 | | 2.03 | | 0.06 | | 0.15 | | 0.12 | | | 0.02 | | 0.02 | | 1.39 | | 0.65 | | | 1.48 | Wang et al., 2019 |
|  | GoM-2 | 3.18 | 29.58 | 11.11 | 221 | 2.47 | 4.98 | 4.13 | 9.57 | | 3.58 | | 280 | | 6.51 | | 0.78 | | 57 | | 2.27 | | 4.69 | | 0.59 | | 2.39 | | 0.52 | | 0.07 | | 0.38 | | 2.89 | | 0.08 | | 0.20 | | 0.18 | | | 0.03 | | 0.02 | | 1.79 | | 0.82 | | | 0.48 | Wang et al., 2019 |
|  | GoM-3 | 0.80 | 11.98 | 4.68 | 128 | 0.76 | 3.39 | 2.67 | 3.45 | | 1.08 | | 2325 | | 0.76 | | 0.26 | | 8 | | 1.57 | | 2.17 | | 0.36 | | 1.45 | | 0.26 | | 0.03 | | 0.16 | | 1.20 | | 0.03 | | 0.08 | | 0.07 | | | 0.01 | | 0.02 | | 0.93 | | 0.23 | | | 1.80 | Wang et al., 2019 |
|  | GoM-4 | 1.43 | 13.71 | 26.48 | 199 | 1.30 | 3.89 | 2.97 | 6.77 | | 1.51 | | 270 | | 2.22 | | 1.48 | | 100 | | 1.79 | | 3.32 | | 0.46 | | 1.87 | | 0.39 | | 0.06 | | 0.31 | | 2.40 | | 0.06 | | 0.17 | | 0.15 | | | 0.02 | | 0.02 | | 1.43 | | 0.52 | | | 1.28 | Wang et al., 2019 |
|  | GoM-5 | 1.95 | 19.85 | 10.82 | 112 | 1.43 | 3.38 | 2.26 | 6.11 | | 1.94 | | 179 | | 2.87 | | 0.09 | | 57 | | 1.96 | | 3.70 | | 0.49 | | 1.98 | | 0.42 | | 0.06 | | 0.33 | | 2.65 | | 0.07 | | 0.18 | | 0.15 | | | 0.02 | | 0.01 | | 1.47 | | 0.51 | | | 0.12 | Wang et al., 2019 |
|  | GoM-6 | 2.20 | 24.58 | 16.41 | 219 | 2.91 | 5.73 | 3.26 | 7.76 | | 2.30 | | 184 | | 3.41 | | 0.13 | | 60 | | 3.06 | | 5.35 | | 0.74 | | 3.06 | | 0.66 | | 0.10 | | 0.56 | | 4.61 | | 0.12 | | 0.31 | | 0.26 | | | 0.04 | | 0.01 | | 2.53 | | 0.67 | | | 0.54 | Wang et al., 2019 |
|  | GoM-7 | 2.21 | 36.44 | 6.36 | 60 | 0.98 | 2.63 | 2.96 | 6.17 | | 2.72 | | 191 | | 3.67 | | 0.46 | | 126 | | 3.30 | | 6.68 | | 0.79 | | 3.10 | | 0.62 | | 0.08 | | 0.45 | | 3.23 | | 0.09 | | 0.24 | | 0.21 | | | 0.03 | | 0.01 | | 1.42 | | 0.83 | | | 1.22 | Wang et al., 2019 |

Table S3. Elemental concentrations for detrital silicate fractions (concentrated HF + HCl leachates) of authigenic carbonates (mg/kg) from the Chukchi Borderlands.

| **Sites** | **Sample ID** | Detrital silicate fractions (conc. HF+HCl) (ppm) | | | | | | |  | |  | |  |  |  | |  | |  | |  | |  | |  | |  | |  |  | |  | |  | |  |  | |  | |  | |  |  | |  | |  | |  |  | Reference |
| --- | --- | --- | --- | --- | --- | --- | --- | --- | --- | --- | --- | --- | --- | --- | --- | --- | --- | --- | --- | --- | --- | --- | --- | --- | --- | --- | --- | --- | --- | --- | --- | --- | --- | --- | --- | --- | --- | --- | --- | --- | --- | --- | --- | --- | --- | --- | --- | --- | --- | --- | --- | --- |
|  |  | Li | Ti | V | Mn | Co | Ni | Cu | Zn | Rb | | Sr | | Zr | Mo | Ba | | La | | Ce | | Pr | | Nd | | Sm | | Tb | | | Dy | | Y | | Ho | | | Er | | Yb | | Lu | | | W | | Pb | | Th | | U |  |
| **Chukchi borderland**  **(CB)** | CB-1 | 57.02 | 4652.94 | 142.26 | 157 | 7.19 | 23.46 | 4.81 | 90.02 | 107.61 | | 142.87 | | 114.21 | 0.86 | 458 | | 21.14 | | 43.76 | | 4.90 | | 17.45 | | 2.81 | | 0.34 | | | 2.32 | | 18.91 | | 0.53 | | | 1.66 | | 1.85 | | 0.27 | | | 1.50 | | 4.80 | | 5.39 | | 2.00 | This study |
|  | CB-2 | 59.00 | 4967.64 | 154.12 | 212 | 7.07 | 24.03 | 4.70 | 85.95 | 123.49 | | 155.10 | | 126.87 | 9.20 | 502 | | 19.88 | | 41.80 | | 4.76 | | 16.84 | | 2.82 | | 0.36 | | | 2.48 | | 20.69 | | 0.56 | | | 1.75 | | 1.97 | | 0.30 | | | 1.62 | | 3.64 | | 5.73 | | 3.10 | This study |
|  | CB-3 | 55.70 | 4817.46 | 116.62 | 182 | 6.35 | 20.03 | 4.74 | 75.07 | 99.88 | | 111.20 | | 134.34 | 1.13 | 410 | | 20.69 | | 44.14 | | 5.15 | | 18.07 | | 3.02 | | 0.38 | | | 2.45 | | 17.53 | | 0.55 | | | 1.71 | | 1.81 | | 0.28 | | | 1.64 | | 4.53 | | 6.15 | | 2.05 | This study |
|  | CB-4 | 68.78 | 4384.58 | 160.75 | 120 | 6.18 | 25.84 | 5.94 | 115.34 | 107.11 | | 108.64 | | 112.09 | 0.71 | 488 | | 22.65 | | 46.31 | | 5.08 | | 17.46 | | 2.68 | | 0.31 | | | 2.10 | | 17.33 | | 0.49 | | | 1.55 | | 1.75 | | 0.27 | | | 1.52 | | 4.99 | | 5.12 | | 2.13 | This study |
|  | CB-5 | 39.99 | 3764.94 | 89.69 | 240 | 6.01 | 18.51 | 4.65 | 91.18 | 77.98 | | 136.00 | | 125.14 | 1.91 | 329 | | 19.70 | | 36.57 | | 4.13 | | 14.80 | | 2.56 | | 0.34 | | | 2.30 | | 18.57 | | 0.52 | | | 1.60 | | 1.74 | | 0.26 | | | 1.24 | | 4.25 | | 4.87 | | 1.75 | This study |
|  | CB-6 | 59.56 | 4614.30 | 121.88 | 115 | 5.25 | 20.94 | 5.98 | 115.95 | 95.24 | | 106.86 | | 142.17 | 0.72 | 431 | | 20.92 | | 43.80 | | 4.97 | | 17.46 | | 2.83 | | 0.36 | | | 2.43 | | 20.12 | | 0.55 | | | 1.72 | | 1.89 | | 0.28 | | | 1.57 | | 5.00 | | 5.59 | | 2.11 | This study |
|  | CB-7 | 62.14 | 4769.26 | 145.82 | 141 | 6.65 | 22.55 | 11.98 | 130.72 | 112.85 | | 438.29 | | 127.76 | 0.89 | 3846 | | 22.52 | | 47.07 | | 5.40 | | 18.84 | | 3.00 | | 0.36 | | | 2.49 | | 20.82 | | 0.58 | | | 1.82 | | 2.04 | | 0.31 | | | 1.57 | | 5.26 | | 5.80 | | 2.31 | This study |
| **Congo Fan**  **(CF)** | CF-1 | 93.60 | 3453.00 | 65.00 | 70 | 6.08 | 24.00 | 12.60 | 45.40 | 70.00 | | 931.00 | | 98.00 | 1.67 | 22659 | | 24.40 | | 35.50 | | 4.20 | | 13.89 | | 2.41 | | 0.30 | | | 1.81 | | 10.94 | | 0.37 | | | 1.09 | | 1.14 | | 0.17 | | | 1.44 | | 9.80 | | 5.41 | | 4.23 | Wang et al., 2019 |
|  | CF-2 | 101.30 | 4052.00 | 74.40 | 64 | 6.50 | 25.80 | 11.50 | 43.80 | 72.00 | | 35.00 | | 125.00 | 1.56 | 257 | | 24.10 | | 34.92 | | 4.16 | | 13.72 | | 2.34 | | 0.33 | | | 1.98 | | 12.89 | | 0.41 | | | 1.23 | | 1.30 | | 0.19 | | | 1.59 | | 10.18 | | 5.59 | | 1.95 | Wang et al., 2019 |
|  | CF-3 | 107.50 | 4421.00 | 80.50 | 68 | 5.83 | 44.40 | 15.30 | 59.00 | 64.00 | | 63.00 | | 135.00 | 4.31 | 396 | | 38.62 | | 55.56 | | 6.56 | | 21.53 | | 3.47 | | 0.39 | | | 2.16 | | 12.10 | | 0.41 | | | 1.16 | | 1.13 | | 0.16 | | | 1.79 | | 17.14 | | 8.52 | | 2.53 | Wang et al., 2019 |
| **Nile Deep-Sea Fan**  **(NDSF)** | NDSF-1 | 52.00 | 7421.00 | 109.00 | 103 | 7.75 | 33.90 | 8.10 | 52.70 | 46.00 | | 163.00 | | 261.00 | 5.69 | 204 | | 28.31 | | 43.31 | | 4.96 | | 16.96 | | 2.91 | | 0.42 | | | 2.76 | | 20.34 | | 0.61 | | | 1.89 | | 2.05 | | 0.30 | | | 1.58 | | 5.86 | | 3.60 | | 1.60 | Wang et al., 2019 |
|  | NDSF-2 | 57.70 | 6905.00 | 123.60 | 228 | 15.93 | 52.20 | 37.20 | 59.20 | 61.00 | | 91.00 | | 213.00 | 24.75 | 209 | | 16.20 | | 25.97 | | 3.05 | | 10.83 | | 2.02 | | 0.35 | | | 2.29 | | 15.97 | | 0.50 | | | 1.54 | | 1.65 | | 0.24 | | | 1.56 | | 8.44 | | 3.94 | | 1.71 | Wang et al., 2019 |
|  | NDSF-3 | 57.60 | 5832.00 | 119.90 | 129 | 7.82 | 34.70 | 15.70 | 47.70 | 62.00 | | 113.00 | | 217.00 | 1.86 | 231 | | 26.90 | | 45.32 | | 5.10 | | 17.82 | | 3.07 | | 0.43 | | | 2.73 | | 18.90 | | 0.59 | | | 1.77 | | 1.91 | | 0.28 | | | 1.21 | | 7.60 | | 6.77 | | 2.14 | Wang et al., 2019 |
|  | NDSF-4 | 41.20 | 4062.00 | 65.00 | 106 | 6.13 | 27.20 | 11.40 | 44.00 | 59.00 | | 1091.00 | | 144.00 | 3.96 | 13128 | | 19.67 | | 32.94 | | 3.73 | | 13.11 | | 2.31 | | 0.33 | | | 2.11 | | 14.54 | | 0.46 | | | 1.39 | | 1.50 | | 0.22 | | | 0.97 | | 7.12 | | 3.61 | | 1.57 | Wang et al., 2019 |
| **Niger Fan**  **(NF)** | NF-1 | n.d. | n.d. | n.d. | n.d. | n.d. | n.d. | n.d. | n.d. | n.d. | | n.d. | | n.d. | n.d. | n.d. | | n.d. | | n.d. | | n.d. | | n.d. | | n.d. | | n.d. | | | n.d. | | n.d. | | n.d. | | | n.d. | | n.d. | | n.d. | | | n.d. | | n.d. | | n.d. | | n.d. | Wang et al., 2019 |
|  | NF-2 | n.d. | n.d. | n.d. | n.d. | n.d. | n.d. | n.d. | n.d. | n.d. | | n.d. | | n.d. | n.d. | n.d. | | n.d. | | n.d. | | n.d. | | n.d. | | n.d. | | n.d. | | | n.d. | | n.d. | | n.d. | | | n.d. | | n.d. | | n.d. | | | n.d. | | n.d. | | n.d. | | n.d. | Wang et al., 2019 |
|  | NF-3 | 109.40 | 5623.00 | 109.00 | 77 | 6.73 | 30.30 | 5.90 | 45.70 | 78.00 | | 92.00 | | 183.00 | 5.19 | 239 | | 51.02 | | 89.50 | | 8.98 | | 29.57 | | 4.63 | | 0.52 | | | 2.98 | | 18.01 | | 0.61 | | | 1.77 | | 1.82 | | 0.27 | | | 1.33 | | 16.61 | | 7.38 | | 1.89 | Wang et al., 2019 |
|  | NF-4 | 68.40 | 3291.00 | 176.80 | 211 | 9.58 | 31.90 | 5.60 | 66.70 | 68.00 | | 52.00 | | 118.00 | 3.31 | 144 | | 35.20 | | 57.90 | | 6.27 | | 20.56 | | 3.26 | | 0.37 | | | 2.09 | | 12.20 | | 0.42 | | | 1.21 | | 1.25 | | 0.19 | | | 2.16 | | 14.64 | | 5.45 | | 1.14 | Wang et al., 2019 |
| **Gulf of Mexico**  **(GoM)** | GoM-1 | 46.50 | 3588.00 | 154.70 | 91 | 4.45 | 16.00 | 5.70 | 42.00 | 102.00 | | 143.00 | | 116.00 | 1.22 | 511 | | 19.11 | | 33.25 | | 3.66 | | 12.65 | | 2.02 | | 0.27 | | | 1.78 | | 12.93 | | 0.41 | | | 1.32 | | 1.48 | | 0.22 | | | 1.01 | | 4.42 | | 4.28 | | 1.95 | Wang et al., 2019 |
|  | GoM-2 | 54.90 | 3065.00 | 120.70 | 98 | 4.37 | 16.80 | 3.50 | 34.70 | 111.00 | | 80.00 | | 109.00 | 1.69 | 497 | | 24.67 | | 45.29 | | 4.92 | | 16.95 | | 2.64 | | 0.34 | | | 2.24 | | 15.39 | | 0.51 | | | 1.59 | | 1.74 | | 0.26 | | | 0.87 | | 4.77 | | 5.37 | | 2.74 | Wang et al., 2019 |
|  | GoM-3 | 49.90 | 3711.00 | 132.90 | 100 | 4.80 | 28.60 | 21.70 | 47.60 | 102.00 | | 125.00 | | 123.00 | 3.12 | 478 | | 24.25 | | 41.85 | | 4.65 | | 16.02 | | 2.56 | | 0.34 | | | 2.23 | | 15.63 | | 0.50 | | | 1.56 | | 1.72 | | 0.25 | | | 1.36 | | 7.65 | | 7.34 | | 2.65 | Wang et al., 2019 |
|  | GoM-4 | 59.50 | 3911.00 | 133.80 | 178 | 6.83 | 29.80 | 12.10 | 58.20 | 120.00 | | 93.00 | | 125.00 | 12.18 | 441 | | 25.05 | | 44.53 | | 4.82 | | 16.55 | | 2.64 | | 0.36 | | | 2.36 | | 16.39 | | 0.53 | | | 1.65 | | 1.79 | | 0.26 | | | 1.39 | | 6.16 | | 5.57 | | 2.24 | Wang et al., 2019 |
|  | GoM-5 | 51.40 | 3360.00 | 110.50 | 117 | 6.03 | 18.50 | 10.30 | 41.30 | 107.00 | | 90.00 | | 129.00 | 1.21 | 547 | | 24.19 | | 45.22 | | 4.87 | | 16.96 | | 2.74 | | 0.38 | | | 2.53 | | 17.14 | | 0.56 | | | 1.73 | | 1.84 | | 0.27 | | | 1.16 | | 6.87 | | 6.35 | | 2.48 | Wang et al., 2019 |
|  | GoM-6 | 53.20 | 4096.00 | 130.60 | 145 | 7.87 | 23.20 | 15.30 | 49.10 | 123.00 | | 97.00 | | 131.00 | 1.81 | 500 | | 24.85 | | 45.51 | | 4.95 | | 17.25 | | 2.80 | | 0.39 | | | 2.57 | | 17.79 | | 0.57 | | | 1.77 | | 1.88 | | 0.28 | | | 1.39 | | 7.03 | | 5.75 | | 2.53 | Wang et al., 2019 |
|  | GoM-7 | 34.00 | 3164.00 | 71.50 | 133 | 4.63 | 15.70 | 6.80 | 30.30 | 76.00 | | 134.00 | | 168.00 | 1.68 | 510 | | 18.61 | | 34.02 | | 3.78 | | 13.44 | | 2.34 | | 0.34 | | | 2.23 | | 15.51 | | 0.50 | | | 1.54 | | 1.65 | | 0.24 | | | 0.80 | | 6.16 | | 4.25 | | 1.67 | Wang et al., 2019 |

n.d. indicates ‘not determined’.

Table S4. Elemental concentrations for total lipids of authigenic carbonates (mg/kg) from the Chukchi Borderlands.

| **Sites** | **Sample**  **ID** | Total lipid fractions (ppm) | | | | | |  | |  | |  | |  | |  | |  | |  | |  | |  | |  | |  | |  |  | |  | |  | |  |  |  | |  | |  | |  |  | |  | |  | |  |  | | Reference |
| --- | --- | --- | --- | --- | --- | --- | --- | --- | --- | --- | --- | --- | --- | --- | --- | --- | --- | --- | --- | --- | --- | --- | --- | --- | --- | --- | --- | --- | --- | --- | --- | --- | --- | --- | --- | --- | --- | --- | --- | --- | --- | --- | --- | --- | --- | --- | --- | --- | --- | --- | --- | --- | --- | --- | --- |
|  |  | Li | Ti | V | Mn | Co | Ni | | Cu | | Zn | | Rb | | Sr | | Zr | | Mo | | Ba | | La | | Ce | | Pr | | Nd | | | Sm | | Tb | | Dy | | Y | | Ho | | Er | | Yb | | | Lu | | W | | Pb | | Th | U |  |
| **Chukchi**  **Borderland**  **(CB)** | CB-1 | 29.94 | 27.34 | 6.70 | 8.54 | 0.55 | 19.70 | | 6.44 | | 103.98 | | 3.13 | | 25.67 | | n.d. | | 0.84 | | 17.50 | | 0.32 | | 0.61 | | 0.07 | | 0.29 | | | 0.03 | | 0.00 | | 0.04 | | 0.27 | | 0.01 | | 0.01 | | 0.03 | | | 0.01 | | n.d. | | 2.06 | | 0.09 | 0.04 | This study |
|  | CB-2 | 26.81 | 13.55 | 2.68 | 27.17 | 0.22 | 4.26 | | 3.02 | | 45.30 | | 2.19 | | 21.03 | | n.d. | | 2.93 | | 13.02 | | 0.24 | | 0.48 | | 0.06 | | 0.19 | | | 0.04 | | 0.00 | | 0.06 | | 0.35 | | 0.02 | | 0.03 | | 0.02 | | | 0.01 | | n.d. | | 1.15 | | 0.09 | 0.77 | This study |
|  | CB-3 | 13.31 | 28.94 | 3.41 | 40.18 | 0.94 | 8.55 | | 9.38 | | 54.86 | | 2.30 | | 11.61 | | n.d. | | 1.08 | | 16.39 | | 0.42 | | 0.96 | | 0.13 | | 0.45 | | | 0.09 | | 0.01 | | 0.07 | | 0.33 | | 0.01 | | 0.01 | | 0.03 | | | 0.01 | | n.d. | | 1.07 | | 0.15 | 0.03 | This study |
|  | CB-4 | 2.80 | 222.31 | 15.41 | 17.81 | 1.93 | 18.35 | | 5.44 | | 64.80 | | 3.03 | | 36.47 | | n.d. | | 0.45 | | 22.34 | | 0.64 | | 1.34 | | 0.18 | | 0.67 | | | 0.14 | | 0.01 | | 0.11 | | 0.49 | | 0.02 | | 0.06 | | 0.03 | | | 0.00 | | n.d. | | 1.56 | | 0.21 | 0.02 | This study |
|  | CB-5 | 6.59 | 110.71 | 5.02 | 59.34 | 8.61 | 26.88 | | 4.48 | | 60.40 | | 4.13 | | 45.05 | | n.d. | | 1.94 | | 20.60 | | 0.55 | | 0.95 | | 0.11 | | 0.69 | | | 0.11 | | 0.02 | | 0.11 | | 0.87 | | 0.03 | | 0.12 | | 0.04 | | | 0.00 | | n.d. | | 4.83 | | 0.21 | 0.15 | This study |
|  | CB-6 | n.d. | n.d. | n.d. | n.d. | n.d. | n.d. | | n.d. | | n.d. | | n.d. | | n.d. | | n.d. | | n.d. | | n.d. | | n.d. | | n.d. | | n.d. | | n.d. | | | n.d. | | n.d. | | n.d. | | n.d. | | n.d. | | n.d. | | n.d. | | | n.d. | | n.d. | | n.d. | | n.d. | n.d. | This study |
|  | CB-7 | 21.88 | 33.39 | 5.64 | 30.60 | 0.99 | 18.06 | | 225.07 | | 300.38 | | 3.90 | | 34.27 | | n.d. | | 1.81 | | 51.97 | | 0.29 | | 0.75 | | 0.10 | | 0.52 | | | 0.09 | | 0.01 | | 0.11 | | 0.47 | | 0.01 | | 0.05 | | 0.05 | | | 0.01 | | n.d. | | 4.17 | | 0.13 | 0.16 | This study |
| **Congo Fan**  **CF)** | CF-1 | 27.90 | 13.04 | 1.41 | 25.00 | 0.22 | 2.18 | | 2.74 | | 13.50 | | 1.40 | | 77.20 | | 0.46 | | 0.42 | | 634.00 | | 0.38 | | 1.00 | | 0.10 | | 0.36 | | | 0.07 | | 0.01 | | 0.05 | | 0.35 | | 0.01 | | 0.03 | | 0.03 | | | n.d. | | 0.04 | | 0.47 | | 0.13 | 0.47 | Wang et al., 2019 |
|  | CF-2 | 21.70 | 16.99 | 2.61 | 8.30 | 0.29 | 2.60 | | 2.38 | | 10.10 | | 1.01 | | 51.90 | | 1.04 | | 0.52 | | 38.00 | | 0.38 | | 0.97 | | 0.09 | | 0.34 | | | 0.07 | | 0.01 | | 0.06 | | 0.43 | | 0.01 | | 0.04 | | 0.04 | | | 0.01 | | 0.04 | | 0.66 | | 0.14 | 0.44 | Wang et al., 2019 |
|  | CF-3 | 10.10 | 17.58 | 1.96 | 5.90 | 0.15 | 1.17 | | 0.94 | | 6.50 | | 0.68 | | 183.00 | | 0.27 | | 0.14 | | 8.70 | | 0.44 | | 0.88 | | 0.09 | | 0.36 | | | 0.06 | | 0.01 | | 0.03 | | 0.20 | | 0.01 | | 0.02 | | 0.01 | | | n.d. | | 0.01 | | 0.51 | | 0.13 | 0.19 | Wang et al., 2019 |
| **Nile Deep-Sea**  **Fan**  **(NDSF)** | NDSF-1 | 17.10 | 26.06 | 1.91 | 15.40 | 0.36 | 1.56 | | 4.48 | | 10.20 | | 0.71 | | 208.00 | | 0.92 | | 0.67 | | 5.40 | | 0.37 | | 0.82 | | 0.09 | | 0.37 | | | 0.07 | | 0.01 | | 0.06 | | 0.32 | | 0.01 | | 0.03 | | 0.02 | | | n.d. | | 0.01 | | 0.27 | | 0.06 | 0.24 | Wang et al., 2019 |
|  | NDSF-2 | 12.90 | 10.14 | 1.10 | 10.00 | 0.38 | 1.65 | | 2.86 | | 11.20 | | 0.57 | | 19.80 | | 0.34 | | 0.20 | | 8.20 | | 0.21 | | 0.57 | | 0.05 | | 0.22 | | | 0.05 | | 0.01 | | 0.04 | | 0.26 | | 0.01 | | 0.02 | | 0.02 | | | n.d. | | 0.02 | | 0.59 | | 0.04 | 0.12 | Wang et al., 2019 |
|  | NDSF-3 | 24.40 | 11.98 | 1.27 | 4.80 | 0.27 | 9.84 | | 37.90 | | 6.80 | | 0.68 | | 153.00 | | 0.24 | | 0.55 | | 8.90 | | 0.19 | | 0.36 | | 0.05 | | 0.20 | | | 0.04 | | 0.01 | | 0.04 | | 0.32 | | 0.01 | | 0.02 | | 0.02 | | | n.d. | | 0.05 | | 0.39 | | 0.03 | 0.11 | Wang et al., 2019 |
|  | NDSF-4 | 16.40 | 3.01 | 0.41 | 3.00 | 0.07 | 1.54 | | 20.50 | | 12.70 | | 0.48 | | 87.60 | | 0.14 | | 0.17 | | 163.00 | | 0.08 | | 0.16 | | 0.02 | | 0.09 | | | 0.02 | | n.d. | | 0.01 | | 0.10 | | n.d. | | 0.01 | | 0.01 | | | n.d. | | 0.01 | | 0.24 | | 0.01 | 0.05 | Wang et al., 2019 |
| **Niger Fan**  **(NF)** | NF-1 | 9.79 | 2.58 | 1.66 | 2.10 | 0.09 | 1.87 | | 3.21 | | 9.30 | | 0.84 | | 507.00 | | 0.14 | | 2.98 | | 13.50 | | 0.06 | | 0.18 | | 0.01 | | 0.07 | | | 0.01 | | 0.00 | | 0.01 | | 0.05 | | n.d. | | n.d. | | n.d. | | | n.d. | | 0.02 | | 0.39 | | 0.01 | 0.30 | Wang et al., 2019 |
|  | NF-2 | 32.60 | 8.23 | 2.08 | 7.00 | 0.33 | 2.73 | | 3.41 | | 23.10 | | 1.01 | | 1106.00 | | 0.26 | | 0.76 | | 28.80 | | 0.66 | | 1.41 | | 0.14 | | 0.59 | | | 0.09 | | 0.01 | | 0.04 | | 0.23 | | 0.01 | | 0.02 | | 0.01 | | | n.d. | | 0.03 | | 0.86 | | 0.06 | 0.99 | Wang et al., 2019 |
|  | NF-3 | 31.30 | 1.14 | 0.72 | 5.90 | 0.40 | 3.10 | | 2.80 | | 7.70 | | 0.80 | | 3.84 | | 0.12 | | 0.16 | | 2.80 | | 0.14 | | 0.38 | | 0.04 | | 0.16 | | | 0.04 | | 0.00 | | 0.03 | | 0.20 | | 0.01 | | 0.02 | | 0.02 | | | n.d. | | 0.02 | | 0.29 | | 0.02 | 0.08 | Wang et al., 2019 |
|  | NF-4 | n.d. | n.d. | n.d. | n.d. | n.d. | n.d. | | n.d. | | n.d. | | n.d. | | n.d. | | n.d. | | n.d. | | n.d. | | n.d. | | n.d. | | n.d. | | n.d. | | | n.d. | | n.d. | | n.d. | | n.d. | | n.d. | | n.d. | | n.d. | | | n.d. | | n.d. | | n.d. | | n.d. | n.d. | Wang et al., 2019 |
| **Gulf of Mexico**  **(GoM)** | GoM-1 | 6.76 | 4.78 | 2.13 | 11.80 | 0.17 | 1.48 | | 3.79 | | 6.30 | | 0.82 | | 221.00 | | 0.26 | | 0.12 | | 8.70 | | 0.20 | | 0.42 | | 0.05 | | 0.19 | | | 0.04 | | n.d. | | 0.03 | | 0.17 | | 0.01 | | 0.01 | | 0.01 | | | n.d. | | 0.02 | | 0.30 | | 0.07 | 0.15 | Wang et al., 2019 |
|  | GoM-2 | 9.03 | 4.61 | 9.30 | 10.50 | 0.22 | 5.39 | | 3.49 | | 46.50 | | 0.83 | | 28.00 | | 0.34 | | 0.14 | | 7.90 | | 0.15 | | 0.35 | | 0.04 | | 0.14 | | | 0.03 | | n.d. | | 0.02 | | 0.12 | | n.d. | | 0.01 | | 0.01 | | | n.d. | | 0.03 | | 0.21 | | 0.04 | 0.03 | Wang et al., 2019 |
|  | GoM-3 | 6.94 | 8.38 | 1.79 | 25.60 | 0.24 | 1.81 | | 3.59 | | 4.00 | | 0.89 | | 410.00 | | 0.32 | | 0.20 | | 12.70 | | 0.35 | | 0.59 | | 0.08 | | 0.33 | | | 0.06 | | 0.01 | | 0.04 | | 0.23 | | 0.01 | | 0.02 | | 0.02 | | | n.d. | | 0.04 | | 0.38 | | 0.08 | 0.39 | Wang et al., 2019 |
|  | GoM-4 | 8.19 | 24.43 | 8.62 | 58.60 | 0.46 | 2.20 | | 2.55 | | 8.10 | | 2.20 | | 104.00 | | 0.99 | | 0.63 | | 34.40 | | 0.65 | | 1.24 | | 0.16 | | 0.60 | | | 0.12 | | 0.01 | | 0.09 | | 0.62 | | 0.02 | | 0.05 | | 0.05 | | | 0.01 | | 0.08 | | 0.62 | | 0.18 | 0.42 | Wang et al., 2019 |
|  | GoM-5 | 6.60 | 29.35 | 6.39 | 33.50 | 0.57 | 3.75 | | 3.35 | | 6.30 | | 2.62 | | 82.00 | | 1.33 | | 0.21 | | 33.70 | | 0.88 | | 1.69 | | 0.21 | | 0.81 | | | 0.16 | | 0.02 | | 0.12 | | 0.81 | | 0.03 | | 0.07 | | 0.06 | | | 0.01 | | 0.02 | | 0.89 | | 0.22 | 0.08 | Wang et al., 2019 |
|  | GoM-6 | 6.94 | 27.91 | 6.12 | 49.60 | 0.72 | 2.65 | | 2.10 | | 4.80 | | 2.43 | | 72.00 | | 1.40 | | 0.19 | | 29.90 | | 1.00 | | 1.86 | | 0.24 | | 0.95 | | | 0.19 | | 0.02 | | 0.17 | | 1.10 | | 0.03 | | 0.09 | | 0.08 | | | 0.01 | | 0.01 | | 0.95 | | 0.24 | 0.21 | Wang et al., 2019 |
|  | GoM-7 | 8.25 | 29.55 | 5.25 | 8.50 | 0.19 | 1.73 | | 7.49 | | 22.20 | | 1.00 | | 33.00 | | 0.44 | | 0.13 | | 19.50 | | 0.40 | | 0.80 | | 0.09 | | 0.34 | | | 0.07 | | 0.01 | | 0.05 | | 0.30 | | 0.01 | | 0.03 | | 0.02 | | | n.d. | | 0.01 | | 0.37 | | 0.08 | 0.09 | Wang et al., 2019 |

n.d. indicates ‘not determined’.

Table S5. Lipid biomarker data for authigenic carbonates from the Chukchi Borderlands.

| **Sites** | **Sample**  **ID** | Irregular hydrocarbons | | | | | | isoprenoid dialkyl glycerol diethers | | | | | | non-isoprenoid dialkyl glycerol diethers | | | | | | fatty acids | | | | | | | | | | Reference |
| --- | --- | --- | --- | --- | --- | --- | --- | --- | --- | --- | --- | --- | --- | --- | --- | --- | --- | --- | --- | --- | --- | --- | --- | --- | --- | --- | --- | --- | --- | --- |
|  |  | Crocetane | | PMI | | PMI:3 | | archaeol | | *sn*-2-hydroxyarchaeol | | *sn*-3-hydroxyarchaeol | | DGD_If | | DGD_IIa | | DGD_IId | | i-C15:0 | ai-C15:0 | C16:1ω7 | C16:0 | C17:0 | C18:1ω9 | C18:1ω7 | C18:0 | C20:0 | C22:0 |  |
|  |  | μg g^-1^dw | ‰ VPDB | μg g^-1^dw | ‰ VPDB | μg g^-1^dw | ‰ VPDB | μg g^-1^dw | ‰ VPDB | μg g^-1^dw | ‰ VPDB | μg g^-1^dw | ‰ VPDB | μg g^-1^dw | ‰ VPDB | μg g^-1^dw | ‰ VPDB | μg g^-1^dw | ‰ VPDB | μg g^-1^dw | | | | | | | | | |  |
| **Chukch**  **borderland**  **(CB)** | CB-1 | 0.01* | n.d. | 0.02 | -65.2 | n.d. | n.d. | 0.02 | -87.6 | 0.02 | -90.6 | n.d. | n.d. | 0.03 | -66.5 | 0.01 | n.d. | 0.01 | n.d. | 0.02 | 0.05 | 0.23 | 0.62 | 0.04 | 0.09 | 0.15 | 0.47 | 0.07 | 0.13 | This study |
|  | CB-2 | 0.03* | -28.5 | 0.03 | -68.4 | n.d. | n.d. | 0.04 | -94.1 | 0.05 | -94.7 | n.d. | n.d. | 0.05 | -54.9 | 0.04 | -77.7 | 0.02 | -80.6 | 0.01 | 0.01 | 0.01 | 0.28 | 0.02 | 0.01 | 0.03 | 0.24 | 0.02 | 0.02 | This study |
|  | CB-3 | 0.02* | -28.5 | 0.02 | -79.2 | n.d. | n.d. | 0.05 | -92.1 | 0.05 | -99.4 | n.d. | n.d. | 0.02 | -56.5 | 0.01 | n.d. | 0.01 | n.d. | 0.01 | 0.02 | 0.08 | 0.22 | 0.01 | 0.04 | 0.06 | 0.12 | 0.09 | 0.01 | This study |
|  | CB-4 | 0.02* | -27.9 | 0.02 | -83.9 | n.d. | n.d. | 0.09 | -85.6 | 0.1 | -87.5 | n.d. | n.d. | 0.06 | -53.7 | 0.04 | -50.3 | 0.03 | -50.6 | n.d. | 0.02 | 0.34 | 0.28 | 0.02 | 0.17 | 0.08 | 0.08 | 0.09 | 0.01 | This study |
|  | CB-5 | 0.01* | n.d. | 0.02 | -60.9 | n.d. | n.d. | 0.03 | -97.4 | 0.03 | -94.1 | n.d. | n.d. | 0.02 | -58.5 | 0.01 | n.d. | 0.01 | n.d. | 0 | 0.01 | 0.04 | 0.21 | 0.01 | 0.07 | 0.05 | 0.11 | 0.05 | 0.02 | This study |
|  | CB-6 | 0.01* | n.d. | 0.02 | -88.2 | n.d. | n.d. | 0.18 | -102.9 | 0.32 | -108.0 | n.d. | n.d. | 0.11 | -78.0 | 0.1 | -84.9 | 0.08 | -88.6 | 0.01 | 0.01 | 0.06 | 0.26 | 0.02 | 0.07 | 0.11 | 0.16 | 0.07 | 0.08 | This study |
|  | CB-7 | 0.05* | -25.4 | 0.04 | -61.5 | n.d. | n.d. | 0.03 | -82.9 | 0.02 | -96.9 | n.d. | n.d. | 0.05 | -60.3 | 0.02 | -60.8 | 0.02 | -65.5 | n.d. | n.d. | 0.03 | 0.09 | 0.01 | 0.04 | 0.06 | 0.04 | 0.07 | n.d. | This study |
| **Congo Fan**  **(CF)** | CF-1 | n.d. | n.d. | n.d. | n.d. | n.d. | n.d. | 0.01 | n.d. | 0.03 | -112.5 | n.d. | n.d. | n.d. | n.d. | n.d. | n.d. | n.d. | n.d. | n.d. | n.d. | n.d. | 0.16 | n.d. | n.d. | n.d. | 0.21 | n.d. | 0.04 | Wang et al., 2019 |
|  | CF-2 | n.d. | n.d. | n.d. | n.d. | n.d. | n.d. | 0.03 | -97.6 | 0.04 | -97.4 | n.d. | n.d. | n.d. | n.d. | n.d. | n.d. | n.d. | n.d. | n.d. | n.d. | n.d. | 0.14 | n.d. | n.d. | 0.02 | 0.2 | 0.02 | 0.02 | Wang et al., 2019 |
|  | CF-3 | 0.03 | -87.8 | 0.25 | -122.1 | n.d. | n.d. | 0.83 | -116.2 | 0.6 | -117.3 | 0.22 | -118.6 | 0.43 | -104.7 | 0.07 | -87.1 | 0.11 | -68.8 | 0.01 | n.d. | 0.01 | 0.64 | 0.05 | 0.01 | 0.01 | 0.88 | 0.02 | 0.03 | Wang et al., 2019 |
| **Nile Deep-Sea**  **Fan**  **(NDSF)** | NDSF-1 | 0.21 | -57.9 | 0.42 | -123.6 | 0.49 | -109.9 | 0.92 | -109.3 | 1.28 | -108.9 | 0.1 | -103.4 | 0.17 | -87.5 | 0.16 | -83.7 | 0.22 | -75.1 | 0.05 | 0.02 | 0.05 | 0.23 | 0.03 | 0.07 | 0.14 | 0.39 | 0.04 | 0.02 | Wang et al., 2019 |
|  | NDSF-2 | 0.08 | -45.9 | 0.1 | -112.5 | n.d. | n.d. | n.d. | n.d. | n.d. | n.d. | n.d. | n.d. | n.d. | n.d. | n.d. | n.d. | n.d. | n.d. | n.d. | n.d. | n.d. | 0.06 | n.d. | 0.01 | n.d. | 0.15 | 0.01 | 0.02 | Wang et al., 2019 |
|  | NDSF-3 | 0.05 | -67.9 | 0.02 | -134.4 | n.d. | n.d. | 0.21 | -122.2 | 0.48 | -116.5 | 0.03 | -118.5 | n.d. | n.d. | n.d. | n.d. | n.d. | n.d. | n.d. | n.d. | 0.01 | 0.22 | n.d. | n.d. | 0.02 | 0.18 | 0.01 | 0.02 | Wang et al., 2019 |
|  | NDSF-4 | 0.02 | -52.1 | 0.03 | -107.7 | n.d. | n.d. | 0.36 | -117.4 | 0.46 | -111.0 | 0.21 | -115.9 | 0.06 | -109.9 | 0.12 | -85.5 | 0.22 | -80.3 | n.d. | 0.01 | n.d. | 0.24 | 0.02 | 0.03 | 0.02 | 0.19 | 0.01 | 0.02 | Wang et al., 2019 |
| **Niger Fan**  **(NF)** | NF-1 | 0.25 | -119.7 | 0.25 | -127.5 | 0.01 | n.d. | 6.97 | -125.3 | 22.13 | -120.1 | n.d. | n.d. | 0.41 | -84.0 | 1.07 | -108.6 | 0.55 | -116.3 | 0.02 | 0.02 | n.d. | 0.14 | n.d. | 0.04 | 0.02 | 0.08 | n.d. | 0.01 | Wang et al., 2019 |
|  | NF-2 | 0.07 | -67.4 | 0.05 | -105.3 | n.d. | n.d. | 0.73 | -104.9 | 1.34 | -103.0 | 0.2 | -105.9 | 0.04 | -92.0 | n.d. | n.d. | n.d. | n.d. | n.d. | 0.01 | n.d. | 0.35 | n.d. | 0.01 | 0.01 | 0.3 | 0.01 | 0.02 | Wang et al., 2019 |
|  | NF-3 | 0.09 | -57.0 | n.d. | n.d. | n.d. | n.d. | 0.09 | -123.8 | 0.23 | -115.7 | 0.02 | -112.4 | n.d. | n.d. | n.d. | n.d. | n.d. | n.d. | n.d. | 0.02 | 0.06 | 0.22 | 0.02 | 0.02 | 0.08 | 0.19 | n.d. | 0.06 | Wang et al., 2019 |
|  | NF-4 | 0.13 | -49.6 | n.d. | n.d. | n.d. | n.d. | n.d. | n.d. | n.d. | n.d. | n.d. | n.d. | n.d. | n.d. | n.d. | n.d. | n.d. | n.d. | n.d. | 0.05 | 0.12 | 0.87 | 0.05 | 0.16 | n.d. | 0.78 | 0.02 | 0.02 | Wang et al., 2019 |
| **Gulf of Mexico**  **(GoM)** | GoM-1 | 0.17 | -50.1 | 0.09 | -102.2 | n.d. | n.d. | 0.31 | -124.6 | 0.68 | -99.8 | 0.04 | -100.4 | 0.12 | -32.5 | n.d. | n.d. | n.d. | n.d. | 0.05 | 0.06 | 0.09 | 1.99 | 0.14 | 0.09 | 0.14 | 1.61 | 0.05 | 0.07 | Wang et al., 2019 |
|  | GoM-2 | 0.25 | -32.3 | n.d. | n.d. | n.d. | n.d. | 0.02 | -87.7 | n.d. | n.d. | n.d. | n.d. | 0.04 | -36.3 | n.d. | n.d. | n.d. | n.d. | 0.03 | 0.03 | 0.06 | 0.89 | 0.12 | 0.1 | n.d. | 0.63 | 0.03 | 0.06 | Wang et al., 2019 |
|  | GoM-3 | 0.13 | -93.9 | 0.11 | -119.8 | n.d. | n.d. | 1.76 | -111.8 | 3.2 | -107.3 | 0.29 | -105.4 | 0.21 | -30.2 | n.d. | n.d. | n.d. | n.d. | 0.05 | 0.03 | n.d. | 0.62 | 0.05 | 0.02 | 0.05 | 0.46 | 0.02 | 0.01 | Wang et al., 2019 |
|  | GoM-4 | 0.15 | -95.5 | 0.14 | -111.8 | n.d. | n.d. | 1.48 | -116.5 | 3.08 | -110.7 | 0.09 | -111.4 | 0.29 | -37.6 | 0.25 | -99.8 | 0.39 | -94.0 | 0.01 | 0.02 | n.d. | 0.78 | 0.05 | 0.03 | 0.06 | 0.63 | 0.03 | 0.02 | Wang et al., 2019 |
|  | GoM-5 | 0.07 | -45.4 | 0.03 | -109.4 | n.d. | n.d. | 0.04 | -121.1 | 0.03 | -122.7 | n.d. | n.d. | n.d. | n.d. | n.d. | n.d. | n.d. | n.d. | 0.02 | 0.02 | n.d. | 0.76 | 0.02 | 0.03 | 0.05 | 0.61 | 0.02 | 0.02 | Wang et al., 2019 |
|  | GoM-6 | 0.06 | -57.0 | 0.04 | -132.9 | n.d. | n.d. | 0.06 | -121.7 | 0.04 | -120.6 | n.d. | n.d. | n.d. | n.d. | n.d. | n.d. | n.d. | n.d. | n.d. | 0.02 | n.d. | 0.55 | 0.03 | n.d. | 0.08 | 0.39 | 0.01 | 0.06 | Wang et al., 2019 |
|  | GoM-7 | 0.06 | -55.3 | 0.03 | -109.6 | n.d. | n.d. | n.d. | n.d. | n.d. | n.d. | n.d. | n.d. | n.d. | n.d. | n.d. | n.d. | n.d. | n.d. | n.d. | 0.02 | n.d. | 0.45 | 0.05 | 0.02 | 0.04 | 0.36 | 0.02 | 0.07 | Wang et al., 2019 |

n.d. indicates ‘not determined’.

* indicates not crocetane but phytane.

Table S6. Microbial diversity in authigenic carbonates from the Chukchi Borderlands.

| Taxanomy· | | Study sites | | | | | | | | | | | | | | | | | | | | | |
| --- | --- | --- | --- | --- | --- | --- | --- | --- | --- | --- | --- | --- | --- | --- | --- | --- | --- | --- | --- | --- | --- | --- | --- |
| Domain | Phylum | Chukchi borderland (Western Arctic) | | | | | | | Gulf of Mexico | | | | | | Nile Deep-Sea Fan | | | Niger Fan | | | Congo Fan | | |
|  |  | CB-1 | CB-2 | CB-3 | CB-4 | CB-5 | CB-6 | CB-7 | GoM-1 | GoM-2 | GoM-3 | GoM-4 | GoM-5 | GoM-6 | NDSF-1 | NDSF-2 | NDSF-3 | NF-1 | NF-2 | NF-3 | CF-1 | CF-2 | CF-3 |
| Archaea | Euryarchaeota | 85.00 | n.d. | 98.94 | 99.98 | 92.38 | 99.41 | 96.67 | n.d. | n.d. | n.d. | n.d. | n.d. | n.d. | 90.78 | n.d. | n.d. | 97.45 | n.d. | n.d. | n.d. | n.d. | n.d. |
|  | Lokiarchaeota | 14.37 | n.d. | 0.00 | 0.00 | 6.12 | 0.45 | 1.48 | n.d. | n.d. | n.d. | n.d. | n.d. | n.d. | 8.69 | n.d. | n.d. | 2.55 | n.d. | n.d. | n.d. | n.d. | n.d. |
|  | Bathyarchaeota | 0.48 | n.d. | 1.06 | 0.00 | 1.05 | 0.05 | 0.67 | n.d. | n.d. | n.d. | n.d. | n.d. | n.d. | 0.15 | n.d. | n.d. | 0.00 | n.d. | n.d. | n.d. | n.d. | n.d. |
|  | Thaumarchaeota | 0.15 | n.d. | 0.00 | 0.00 | 0.46 | 0.04 | 1.17 | n.d. | n.d. | n.d. | n.d. | n.d. | n.d. | 0.00 | n.d. | n.d. | 0.00 | n.d. | n.d. | n.d. | n.d. | n.d. |
|  | Unclassified archaea | n.d. | n.d. | n.d. | 0.02 | n.d. | 0.04 | n.d. | n.d. | n.d. | n.d. | n.d. | n.d. | n.d. | 0.38 | n.d. | n.d. | 0.00 | n.d. | n.d. | n.d. | n.d. | n.d. |
| Bacteria | Firmicutes | n.d. | n.d. | n.d. | n.d. | n.d. | 0.66 | n.d. | 98.28 | 20.17 | 87.13 | 88.21 | 49.17 | 64.60 | 1.77 | 88.53 | 62.94 | 4.27 | 19.17 | n.d. | 1.44 | 9.31 | 5.46 |
|  | Proteobacteria | n.d. | n.d. | n.d. | n.d. | n.d. | 7.58 | 11.49 | 1.21 | 19.19 | 10.37 | 10.00 | 36.71 | 30.20 | 30.15 | 9.68 | 26.25 | 20.40 | 35.79 | n.d. | 74.52 | 65.20 | 56.11 |
|  | Atribacteria | n.d. | n.d. | n.d. | n.d. | n.d. | 61.38 | 77.85 | 0.00 | 0.15 | 0.03 | 0.00 | 0.00 | 0.00 | 2.07 | 0.00 | 1.32 | 0.71 | 0.00 | n.d. | 5.77 | 1.47 | 2.18 |
|  | Actinobacteria | n.d. | n.d. | n.d. | n.d. | n.d. | 7.04 | 2.73 | 0.26 | 25.30 | 0.11 | 0.59 | 7.80 | 3.06 | 3.94 | 1.33 | 2.54 | 2.70 | 24.10 | n.d. | 0.00 | 4.90 | 3.06 |
|  | Bacteroidetes | n.d. | n.d. | n.d. | n.d. | n.d. | 2.18 | 0.46 | 0.15 | 10.68 | 1.45 | 0.74 | 2.60 | 1.37 | 2.39 | 0.22 | 1.69 | 31.54 | 6.78 | n.d. | 8.65 | 15.93 | 6.77 |
|  | Planctomycetes | n.d. | n.d. | n.d. | n.d. | n.d. | 4.80 | 5.56 | 0.04 | 3.85 | 0.33 | 0.00 | 1.20 | 0.00 | 12.53 | 0.08 | 0.75 | 4.02 | 0.06 | n.d. | 2.88 | 0.98 | 6.77 |
|  | WM88 | n.d. | n.d. | n.d. | n.d. | n.d. | 0.12 | 0.00 | 0.00 | 0.20 | 0.03 | 0.00 | 0.00 | 0.00 | 11.19 | 0.02 | 1.03 | 21.06 | 0.00 | n.d. | 0.00 | 0.00 | 3.28 |
|  | Others | n.d. | n.d. | n.d. | n.d. | n.d. | 16.23 | 1.91 | 0.06 | 20.46 | 0.56 | 0.45 | 2.51 | 0.77 | 35.97 | 0.13 | 3.48 | 15.29 | 14.10 | n.d. | 6.73 | 2.21 | 16.38 |

n.d. indicates ‘not determined’.


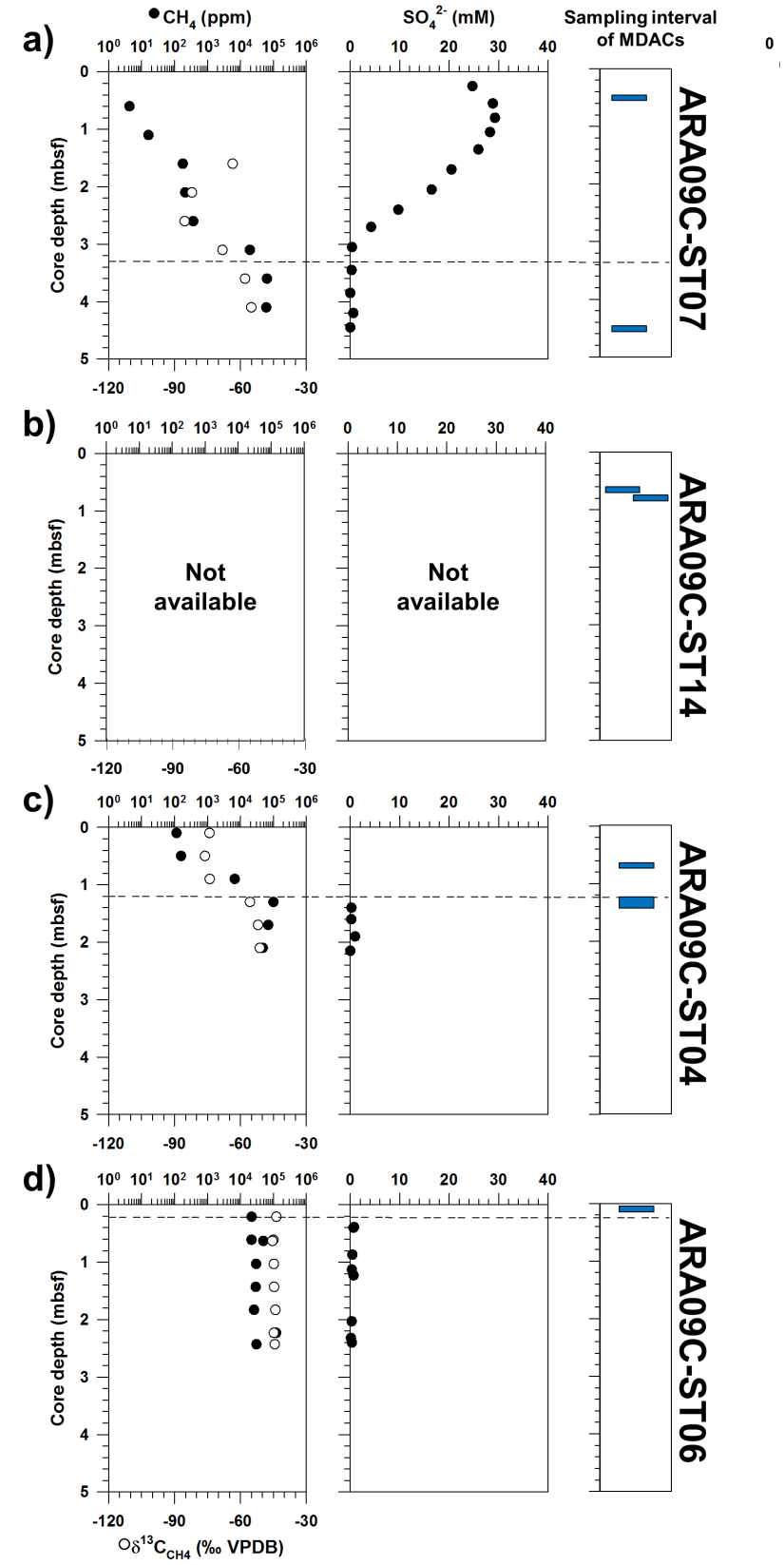


Figure S1. Downcore profiles of CH_4_ concentrations (ppm) and δ^13^C_CH4_ values (‰ VPDB) of headspace gases and dissolved sulfate (mM) in porewater with the indication of the MDAC sampling interval at sites a) ARA09C-ST07, b) ARA09C-ST014, c) ARA09C-ST04, and d) ARA09-ST06 (Kim et al., 2020 and 2022). Note that the data for ARA09C-ST014 are not available. Black dash lines indicate the depths of sulfate-methane transition zone (SMTZ).


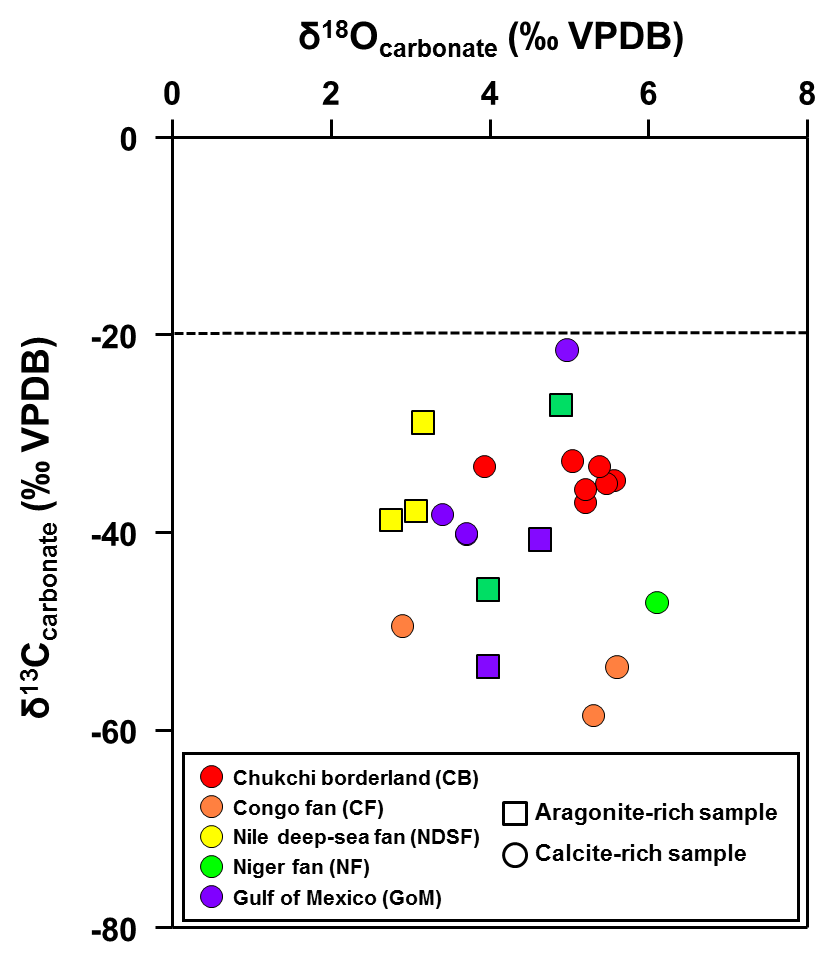


Figure S2. Scatter plot of δ^18^O_carbonate_ and δ^13^C_carbonate_ of authigenic carbonate samples considered in this study. The dashed line indicates the δ^13^C baseline between marine- and methane-derived carbonates.


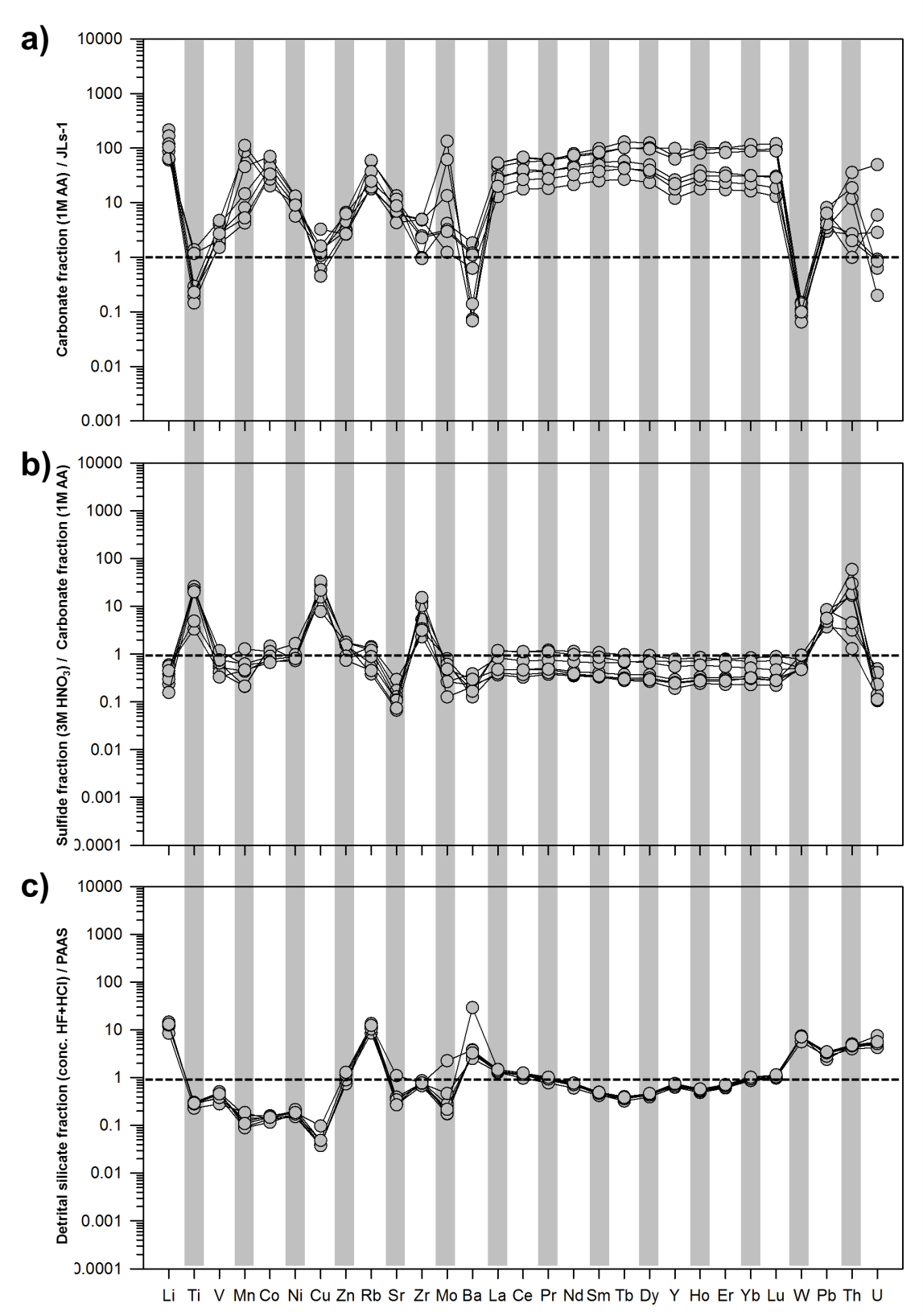


Figure S3. Enrichment factors of a) carbonate fraction (1M AA leachates) data normalized to values for the JLs-1 carbonate reference material, b) sulfide fraction (3M HNO_3_ leachates) data normalized to values for corresponding carbonate fractions (1M AA leachates), and c) detrital silicate fraction (concentrated HF + HCl leachates) data normalized to values for the PAAS reference material.


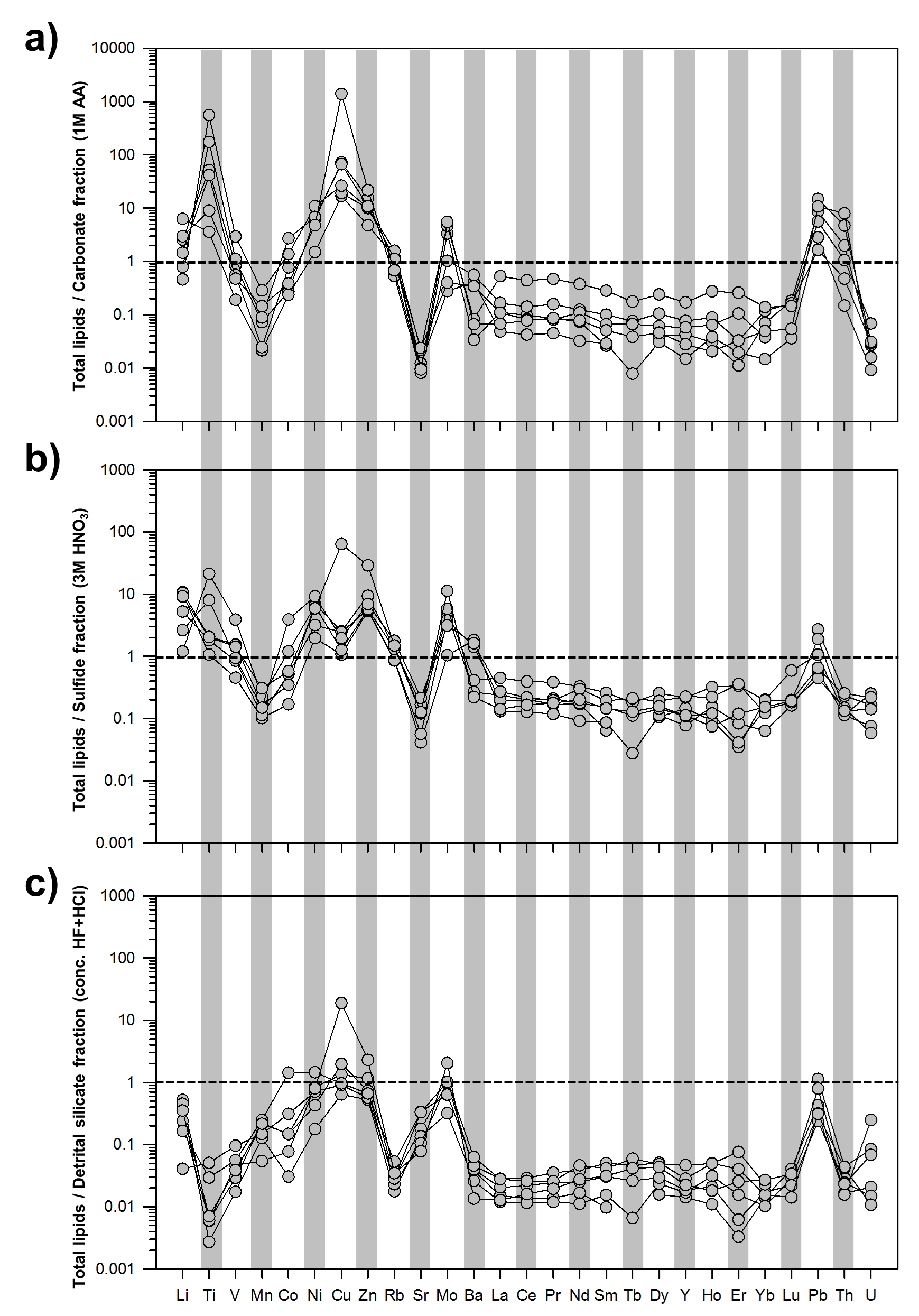


Figure S4. Enrichment factors of total lipids normalized to values for a) carbonate fractions (1M AA leachates), b) sulfide fractions (3M HNO_3_ leachates), and c) detrital silicate fractions (concentrated HF + HCl leachates).


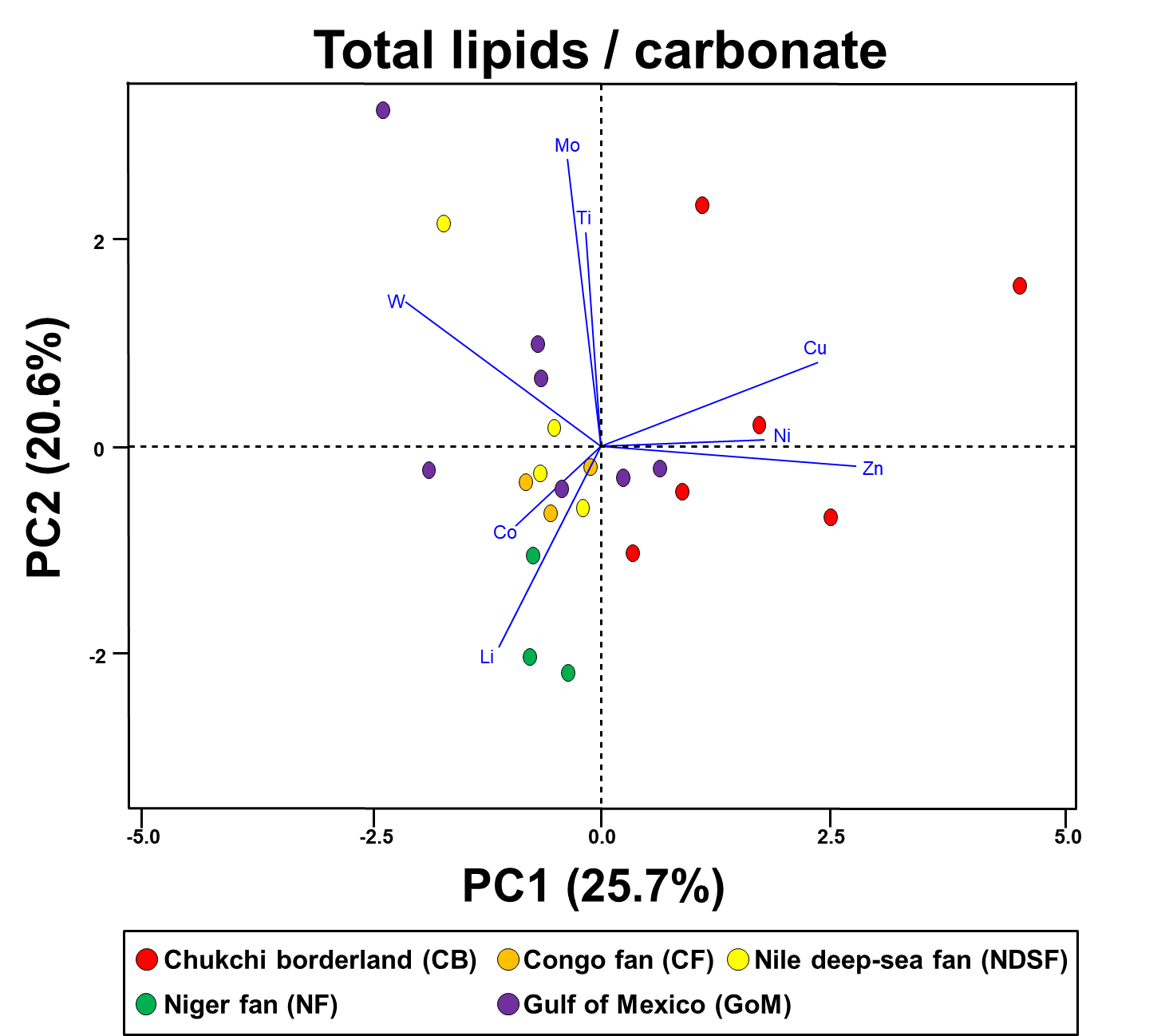


Figure S5. PCA results for the trace metal data for the total lipids normalized to the values of the carbonate fraction.
